# Emotional Competences in Adolescents Exposed to Colombian Armed Conflict During Their Childhood

**DOI:** 10.1007/s40653-024-00647-0

**Published:** 2024-07-30

**Authors:** Diego Armando León-Rodríguez, Catalina Moncaleano

**Affiliations:** 1 Universidad del Externado, Bogotá, Colombia; 2 Pontificia Universidad Javeriana, Bogotá, Colombia

**Keywords:** Armed-conflict, Childhood Adversity, Emotional Competences, Adolescents, Emotional Development, Mediational Model

## Abstract

Objective: Analyse the mediation role of emotional competences on behavior problems in adolescents exposed to Armed-Conflict Childhood Adversities (ACCA). Method: Families with adolescent children who studied in three schools in the municipality of Soacha, Colombia, were invited to participate in the study. One hundred and sixty-one participants were selected and pooled into three groups according to their Adverse Childhood Experiences (ACE) scores: lower-ACE (LACE), higher-ACE (HACE), and ACCA using the Adverse Childhood Experiences Questionnaire for Adolescents (ACE-QA). The Emotion Recognition Task, the Empathy for Pain Task, and the Child Behavior Checklist assessed teenagers’ emotional functioning. Results: Teenagers exposed to ACCA more frequently experienced childhood adversities such as domestic violence, child abuse, and parental neglect. Moreover, these adolescents were less accurate in discriminating angry faces, reported higher control perception after social stressors, were more rigorous in punishing those who intentionally harmed others, and showed more internalizing behavior problems. In the mediational model, we found that: the control perception after social stress mediated the reduction of depressive symptoms in adolescents with ACCA history. Additionally, inaccuracy in perceiving angry faces and harsh punishment toward behaviors that intentionally harm others mediated the presence of thinking problems, anxiety, and rule-breaking behaviors. Conclusions: Colombian adolescents who experienced ACCA showed specific changes in their emotional competences, which mediate the parental report of behavioral problems. These results indicate the need for more focused interventions aimed at improving the emotional competences and mental health of adolescent victims of armed conflicts.

**Supplementary Information:**

The online version contains supplementary material available at 10.1007/s40653-024-00647-0.

## Introduction

Armed conflict (AC) is a frequent worldwide condition, with children being the primary victims. One and a half billion children live in an AC zone surrounded by extreme violence (Jordans et al., 2016; Østby et al., 2020). It is estimated that one in five children is a victim of AC, undergoing abiding and pervasive exposure to several adversities, mainly abuse, death threats, loss of relatives, forced displacement, combat, malnutrition, amputations, and witnessing torture, terrorism, and murders (Save the Children, 2019). Usually, these *Armed-Conflict Childhood Adversities* (ACCA) go hand in hand with other *Adverse* C*hildhood Experiences* (ACE) such as abuse, domestic violence, poverty, parental neglect, and parents’ handicaps. The accumulation of ACCA and ACE affects the neural mechanisms implicated in the development of competences to perceive, control, and understand emotions, increasing the risk of exhibiting behavioral problems during adolescence (Betancourt et al., 2013; Feldman & Vengrober, 2011; Kadir et al., 2019; León-Rodríguez & Cardenas, 2021; McLaughlin et al., 2019).

Some studies have linked armed conflicts with changes in the competence to recognize emotions; for instance, Russian children exposed to terrorism and young survivors of the Sierra Leone war were more sensitive to anger expressions, mistaking other emotional displays (Scrimin et al., 2009; Umiltà et al., 2013). Similarly, Israeli and North American veterans were primed to identify angry faces (Anaki et al., 2012; Ashley & Swick, 2019). Moreover, the skill to manage emotions can be different in people living in AC; often, people surviving wars report exhibiting more frequent and intense distress, anxiety, sadness, and anger (Campo-arias et al., 2017; Çeri & Nasiroğlu, 2018; Cerquera-Córdoba et al., 2020; Roupetz et al., 2020; Slone & Mann, 2016). This negative mood has been related to neuroendocrine dysregulations in response to social complexes (Dajani et al., 2018; Pesonen et al., 2010; Shaheen et al., 2020). Cardona-Isaza & Díaz-Posada (2021) found that Colombian young victims of AC showed problems empathizing with and understanding the feelings of others. Moreover, Colombian ex-combatants showed less personal distress toward others’ pain (Baez et al., 2019; Tobón et al., 2016). Despite all this evidence of the effects of AC on emotional competences, no study has systematically investigated the relationship between AC exposure during childhood and the adolescent’s competences to perceive, respond, understand, and empathize with others’ emotions.

These emotional changes in teens with ACCA history could lead to behavioral problems (DiGangi et al., 2017; Hewitt-Ramírez et al., 2020). Adolescents living through ACCA have a higher risk of developing post-traumatic stress, anxiety, depression, drug abuse, and suicidal ideation, which could last until adulthood, reducing life quality and life expectancy (Joshi & O’Donnell, 2003; Layne et al., 2010). For example, Colombians who lived through multiple ACCAs displayed higher levels of distress, sadness, anxiety, and maladaptive coping skills (Campo-Arias et al., 2017; Cerquera-Córdoba et al., 2020). Moreover, Kenyan children exposed to war experiences were more aggressive and violent (Kithakye et al., 2010). Some of these problems can be sex-dependent, with female adolescents more likely to exhibit internalizing disorders, and male adolescents at higher risk of externalizing problems (Betancourt et al., 2013; Çeri & Nasiroğlu, 2018). However, it is not clear how the effect of ACCA on adolescents’ emotional competences could impact their mental health.

More than two million Colombian children have been victims of AC and often migrate to cities and suburbs (Registro Único de Víctimas [RUV], 2022). Soacha is a municipality next to Bogotá with a population of 660,179 inhabitants and more than fifty thousand refugees from the Colombian AC (Departamento Administrativo Nacional de Estadística [DANE], 2018). These migrants are exposed to other ACEs like domestic violence, abuse, extreme poverty, parental drug use, and neighborhood violence. The accumulation of ACE and ACCA can be associated with typical behavioral problems in Soacha’s teens, like dropping out of school, gang affiliation, drug use, violent behavior, early pregnancy, and suicide (El Espectador, 2022). Therefore, this municipality is an ideal place to understand the impact of ACCA on adolescents’ emotional competence development, which allows us to provide timely interventions to improve their well-being.

To summarize, previous research points out that AC changes emotional functioning and increases mental health issues, but so far, there are no investigations on the relationship between experiencing AC during childhood and competences of perceiving, regulating, and empathizing with others’ emotions in adolescence. Particularly, it is unclear if ACCA causes specific changes in the emotional competences differing from those caused by *low or high childhood adverse experiences (LACE and HACE)*. Therefore, this paper adds new knowledge to the literature by examining the specific changes ACCA makes on the development of emotional competences compared to other kinds of childhood adversity and how these changes can mediate mental health during adolescence. Understanding these specific effects can used to create more efficient and effective intervention strategies for training emotional competencies with adolescents screened for ACEs and ACCA in vulnerable communities.

**Fig. 1:**
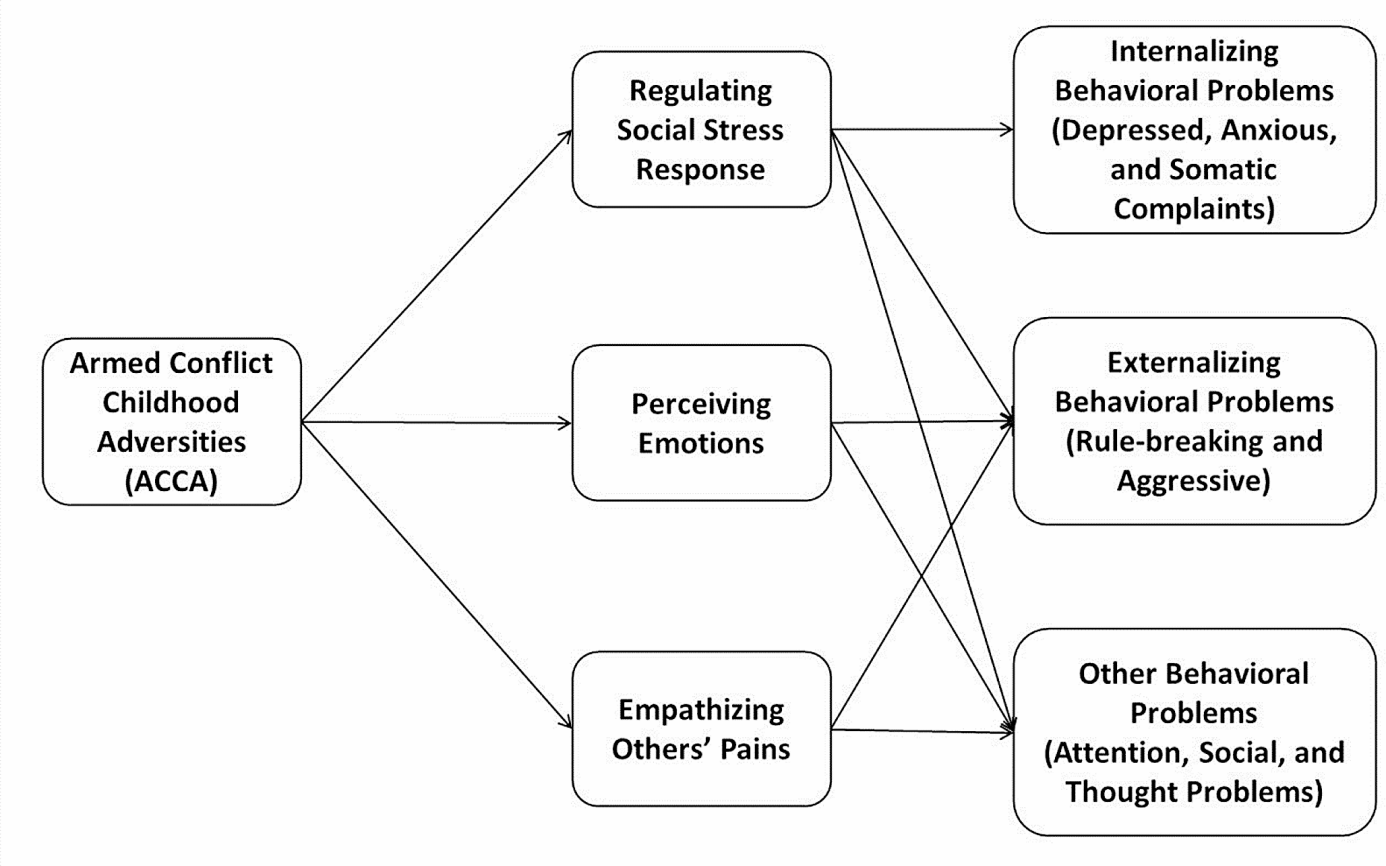
Proposed chain mediational model

This study had five objectives: the first was to detect cases of exposure to ACCA in a group of adolescents from the municipality of Soacha, Colombia; the second was to characterize the childhood adversities reported among ACCA, LACE, and HACE adolescents; the third was to compare among ACCA, LACE, HACE groups the emotional competences to recognize facial emotion expression, respond to social stressors, and empathize to others’ pain; the fourth objective was to compare the parental report of behavioral problems among ACCA, LACE, and HACE teens; and the last objective was to explore in a chain mediational model (as shown in Fig. 1) what is the role of competences to perceiving, regulating, and empathizing with others’ emotions on the report of behavioral problems in adolescents who have experienced childhood armed-conflict. Specifically, we expected that: ACCA would affect social stress regulation, leading to an increase in all behavioral problems; ACCA would lead to failures in emotional perception, causing social and externalizing problems; and ACCA would modify empathic processing, leading to aggressive and antisocial behaviors.

## Method

### Instruments

#### ***Sociodemographic Survey***

This survey inquired 16 questions about sociodemographic health status, drug and medication use, relationships with caregivers, and psychosocial risks.

#### ***Montreal Cognitive Assessment (MOCA)***

This is a brief cognitive screening test validated in adolescents with a sensitivity of 75%, a specificity of 90%, and a reliability of 0.89 in the detection of cognitive deficits (Pike et al., 2017). The maximum score is 30; a score equal to or higher than 26 is considered normal; therefore, adolescents with less than 25 points were excluded from the study.

#### ***The Adverse Childhood Experiences Questionary for Adolescents (ACE-QA)***

It contains forty-four retrospective items inquiring about the frequency of threatful events during childhood. The complete scale had an acceptable internal reliability coefficient (ω = 0.880). ACE-QA is composed of the following nine factors: domestic violence, maltreatment, parental neglect, social loss, health problems, school stress, socioeconomic resources, neighborhood violence, and armed conflict (ACCA). ACCA contained seven items asking about the frequency with which participants experienced painful events related to Colombian armed conflict before age 12, with a range score between 0 and 28. The McDonald’s omega for this subscale in our study was 0.878, showing acceptable internal reliability.

#### ***Facial Emotion Recognition Task (FERT)***

It is a widely used and validated computer task to assess the skills to discriminate facial expressions of emotions (Kessels et al., 2013; Williams et al., 2022). FERT consists of 48 trials, eight for each prototypical emotion (anger, disgust, fear, happiness, sadness, and surprise) presented in E-Prime software. Each trial consisted of 20 images, which displayed a 5% range, from neutral (0%) to complete expression (100%), and it lasted 10 s. Trials were randomly presented, and participants had to discriminate the emotion as soon as possible. Participants were trained to respond as fast as possible with the keyboard, and the software recorded the response’s accuracy and latency.

#### ***Trier Social Stress Test for Adolescents (TSST-A)***

This task is an adolescent-adapted version of the classical TSST, which is a well-validated and widely used procedure to elicit stress responses (Viola et al., 2014). The test consists of an evaluative and uncontrollable social interaction with two interviewers who press the participant to do verbal and arithmetic tasks. The emotional response was assessed on two scales: (1) the Self-Assessment Manikin (SAM), which is a picture-oriented questionnaire that measures valence, arousal, and control dimensions of emotional response; (2) the State Anxiety Inventory for Adolescents (STAI-A) is a Likert questionnaire with 20 items about the current anxiety-related state. Each scale was applied before the social stressors (pre-stress), immediately after the social stressors (post-test), and fifteen minutes after the social stressors (recovery).

#### ***Empathy for Pain Task (EPT)***

It consists of a computer measure of affective and moral components of empathy that is widely validated (Decety et al., 2012). EPT contained 25 videoclips or trials showing damage caused by others in three situations: eleven Intentional Painful Situations (IPS), eleven Accidental Painful Situations (APS), and three Non-Pain Situations (NPS). After each video, participants should respond to five questions to assess the affective and moral pain empathy components. Questions about empathic concern and disconfirm assessed the affective components, while correctness perception and punishment assignment corresponded to the moral dimensions of empathy. Each question was answered on a visual analog scale (-9 to + 9) with the keyboard. This task has been used with the Latin American population (Baez et al., 2018) and was validated for Colombian adolescents (Gutiérrez-Quintana, 2021).

#### ***Child Behavior Checklist (CBCL)***

It is an instrument of 113 Likert-type items that allows the evaluation of children’s behavior based on their parents’ reports. This instrument assesses externalization problems (aggressive behavior, rule-breaking, and attentional problems) and internalization problems (complaints of health problems, isolation, anxiety, and depression). The health information was used to exclude participants with mental or medical diseases. The CBCL is an extensively used caregiver questionnaire to screen for emotional, behavioral, and social problems in children and adolescents. This instrument has already been used in adolescents exposed to Colombian armed conflict (Hewitt-Ramírez et al., 2020). The McDonald’s omega was 0.87 for the complete scale, 0.78 for internalizing problems and 0.77 for externalizing problems subscales.

**Fig. 2:**
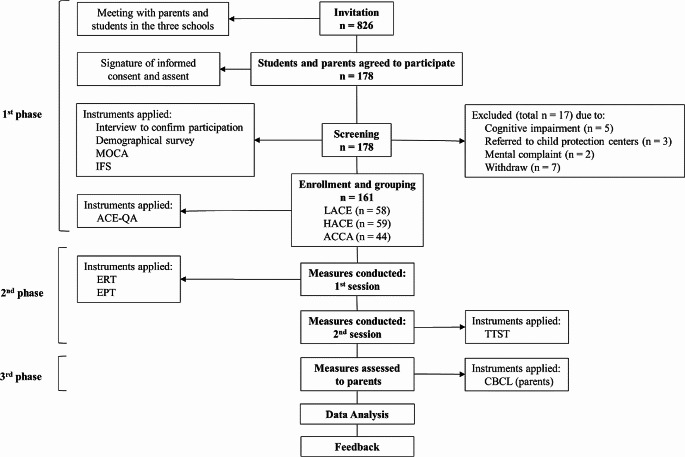
Study moments. *Note.* Montreal Cognitive Assessment (MOCA); INECO Frontal Functions Scale (INF); Adverse Childhood Experiences Questionary for Adolescents (ACE-QA); Emotion Recognition Task (ERT); Empathy for Pain Task (EPT); Trier Social Stress Task (TSST); Child Behavior Checklist (CBCL)

#### Procedure

As shown in Fig. 2, there were three main phases during the development of the study, and all procedures were achieved in schools. The first phase was aligned with research goals one and two. During this phase, 826 families from three schools in Soacha (Colegio Maria Magdalena, Gimnasio la Alameda y Instituto Psicopedagogico Juan Pablo II) were invited to participate in the study. In each of the schools, a meeting was held with parents in which they were informed about the characteristics of the project, the instruments used and the ethical considerations. At this meeting, the parents signed the informed consents and answered the CBCL. Then, an individual meeting was held with each student to confirm their interest in participating and answer the screening instruments: the sociodemographic survey, MOCA, and IFS. After that, selected participants answered the ACE-QA to be assigned to a group. This phase aimed to detect cases of exposure to armed conflict situations, assign groups, and compare childhood experiences.

The second phase was aligned with the research goals three to seven. During this phase, measures were assessed in two sessions of one hour each, following rigorous ethical protocols to provide psychological first aid and protect the well-being of adolescents. In session one, participants individually performed the Emotion Recognition Task (ERT) and Empathy for Pain Task (EPT). In session two, adolescents make the adolescent version of the Trier Social Stress Task (TSST). Finally, the third phase was aligned with the 5th research goal, and parents answered the Child Behavior Checklist (CBCL).

#### Participants

As shown in Figs. 2 and 826 students and their families from three schools in Soacha were invited to participate in the Adverse Childhood Experiences project. Of the invited families, only 178 agreed to participate; students and parents signed the informed consent and assent. fter the screening, 161 participants were left and divided into three groups according to their score in ACE-QA. The ACCA group was composed of those participants exposed to any Armed Conflict Childhood Adversity. The other groups were composed of those adolescents who had not been exposed to armed conflict adversities; participants scoring below the 66th percentile in ACE-QA were included in the Low Childhood Adversity Experiences (LACE) group, and teens above these points of cut were included in the High Childhood Adversity Experiences (HACE) group. The division was done to observe the specific effects of exposure to armed conflict and separate them from the effects that exposure to other kinds of adversities may have.

#### Ethical Considerations

The ethical committee of the Facultad de Ciencias Sociales de la Universidad de los Andes endorsed this study. All procedures satisfied the requirements for underage human research with minimum risk and were used in previous research without adverse effects. The participants and legal representatives were informed about the objectives, risks, and benefits. Each participant received an identification code to protect their privacy. The databases have been under custody to guarantee the security of the information. Psychological tests were applied by psychologists trained in psychological first aid and emotional deactivation. Adolescents’ participation and permanence were free and voluntary.

The families received an individualized summary of the results obtained by each participant and were also invited to workshops to promote parenting practices and socio-emotional development. Whenever indicators of emotional, intellectual, social, or neurological problems were found, parents were personally informed and provided: emotional support, explanations about the problem, guidance on where to get help and treatment, and advice on school adjustments. On the other hand, each school received a report containing a characterization of the participating students. In addition, counseling and training were provided on the specific interventions that would promote the development of socio-emotional skills in the educational community.

Subsequently, given that the study population comprised victims, meticulous attention was given to prevent re-victimization. To achieve this, psychologists involved in the research were trained to provide socio-emotional support and to refrain from using the experiences of the victims solely as information.

#### Data Analysis

Prior power analysis for repeated measures ANOVAs with three groups and six measures from a sample size of 150 participants were enough to get an effect size of 0.25 with an error of 0.05. Descriptive statistics of all dependent measures were calculated including mean, standard deviations, standard error of the mean, median, Shapiro-Wilk normality indicator, and Levene’s test for homogeneity of variances. These analyses are presented in supplementary tables S1, S2, and S3. Exposure to adverse childhood experiences was compared between the ACCA, LACE, and HACE groups. Repeated measures ANOVAs were performed for FERT, TSST-A, and EPT, being between-subject factors in the *childhood experience group* (ACCA, HACE, and HACE) and *sex* (youth-females and youth-males). For FERT, the repeated measures were the *six basic emotions*, and the accuracy and latencies of facial expression recognition were the dependent measures. For the TSST-A, the repeated measures were TSST-A *moments* (pre-stress, post-stress, and recovery), and the three dimensions of SAM (valence, arousal, and control) and the STAI-C scores were dependent measures. In the EPT analyses, the repeated measures were the *pain situation* (NPS, IPS, and APS), and the dependent measures were empathic ratings and latencies. Finally, to analyze the group’s effect on specific emotional competences a variance analysis was run for each emotion, affective dimension of the stress response, and empathic response. When the homogeneity and sphericity assumptions were violated, the *Greenhouse-Geisser* and *Brown-Forsythe* corrections were achieved. For nonparametric distributions, *Kruskal-Wallis* was used. In *post hoc* analyses, corrections for multiple comparisons were made using the *Bonferroni* and *Dunn* procedures; effect sizes were calculated using *Cohen’s d*, and confidence intervals were computed for 95%.

To compute the mediation model, we followed the next procedure. First, seven models were constructed with hypothetical mediational relationships (Table S4, supplementary material). In six of these models, ACCA exposure was the predictor variable with two levels (non-exposure = 0 and exposure = 1), and in one, gender was added. Similarly, in six of these models, emotional competencies were used as mediators and behavioral problems as outcomes; only in one model was this order reversed to test whether behavioral problems could predict emotional functioning (model 5 in Table S4 of the supplementary material). Based on previous analyses, only those emotional responses that had shown a relationship with ACCA were chosen as mediators; not all responses in the emotional competency tasks were included as mediators because this large number of measures saturated the processing capacity of the statistical program. Next, model comparison was conducted using structural equation modeling (SEM), whereby the model showing the best fit was selected. With the best-fitting model, a mediation analysis was run to test the significance of the effects and pathways using a bootstrap method with 1000 replications, and confidence intervals were computed using the bias-corrected percentile method (Biesanz et al., 2010). All statistical procedures were achieved with the software Jeffreys’s Amazing Statistics Program (JASP, 2021).

## Results

### Objective One: Soacha Adolescents’ War Exposure

Table 1 shows the main descriptive results: 27.3% of Soacha’s adolescents reported war experience during their childhood, scoring 11.2 on the ACCA scale. The high standard deviation indicates that the frequency and number of these events varied widely among adolescents exposed to armed conflict.

**Table 1:** Descriptive statistics in LACE, HACE, and ACCA groups

Variable	LACE	HACE	ACCA	All
*N* (%)	58 (36%)	59 (36.7%)	44 (27.3%)	161 (100%)
Girls (% within sex)	39 (67.2)	32 (54.2)	28 (65.9)	100 (62.1)
Age (SD)	15.3 (1.6)	15.4 (1.3)	15.2 (1.4)	15.3 (1.4)
ACE Score (SD)	14.4 (4.6)	53.8 (10.2)	50.7 (23.3)	38.8 (23)
ACCA Score (SD)	0	0	11.2 (5.0)	3.01 (5.6)
Number of ACEs (SD)	4.8 (1.7)	7 (0.9)	7.9 (1.1)	6.4 (1.8)

*Note.* The table shows descriptive information for adolescents’ LACE, HACE, and ACCA groups. First, the sample size (N) and its percentage are presented. Then the number and percentage of girls in each group and the number and percentage of boys can be computed from girls’ data. Next are presented the average and standard deviation (SD) of age, *adverse childhood questionnaire* (ACE score), *armed-conflict sub-scale* (ACCA), and the number of *childhood adversities* (ACEs).

### Objective Two: Childhood Adversity Between Groups

**Fig. 3:**
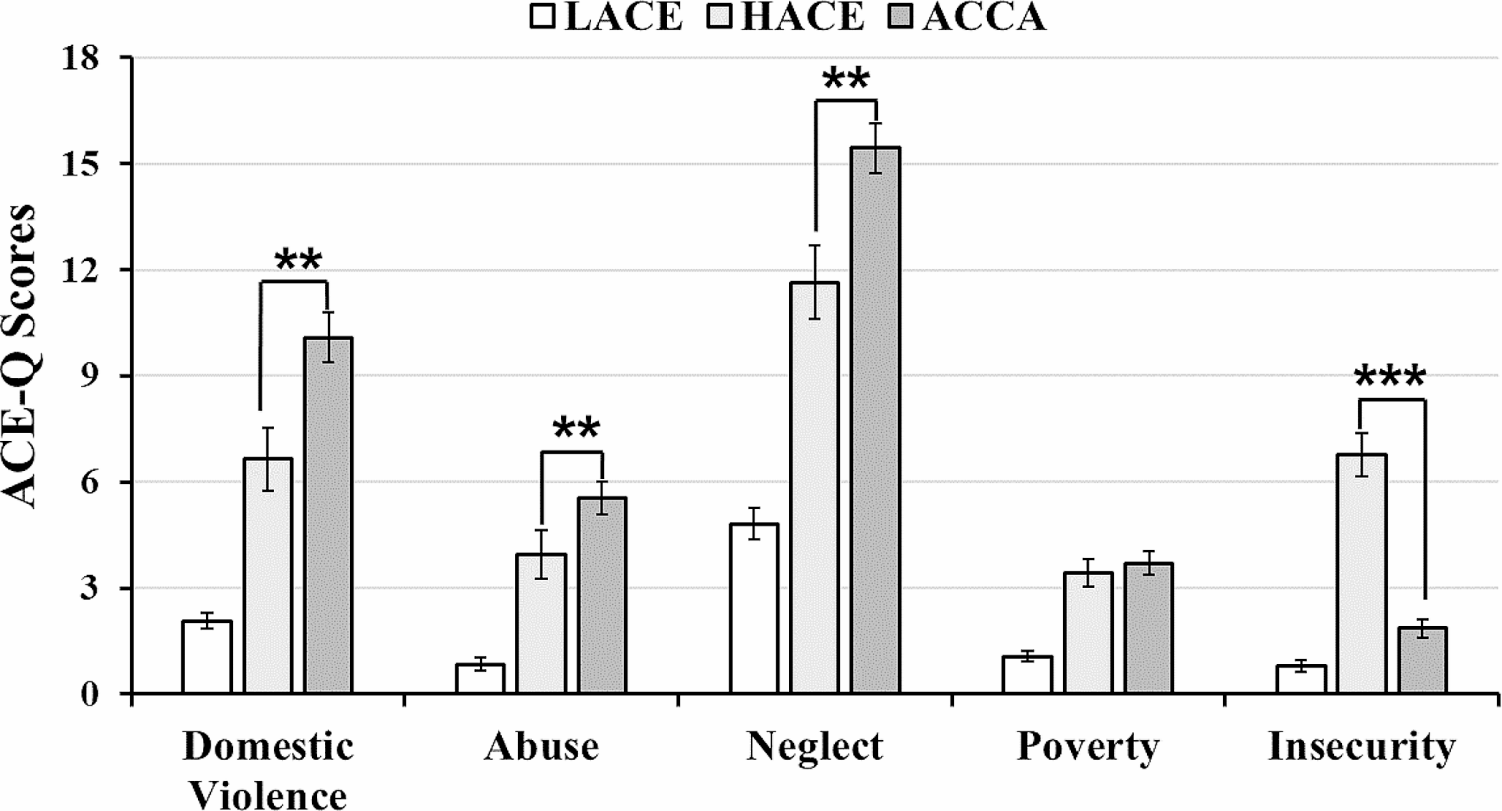
ACE-Q scores for LACE, HACE, and ACCA groups. *Note.* Significant differences are p-values of ***p˂0.001, **p˂0.01

Kruskal-Wallis test for ACE-QA confirmed *childhood experience* differences between groups (χ^2^(2, 152) = 112.17, *p* < 0.001), where LACE adolescents experienced lesser childhood adversities than other teens, while HACE and ACCA groups experienced a high number of early adversities (Table 1). Even though the LACE group had lower ACE-QA scores, they reported an important quantity of early adversities. Teens exposed to ACCA underwent the highest rate of domestic violence, abuse, and neglect (Fig. 3). Repeated measures ANOVA for ACE-QA sub-scales resulted in significant differences between groups (*F*(2, 14) *=* 15.01, *p˂*0.001). Figure 3 shows these differences, *post hoc* comparisons probed that war-exposed teens suffered significantly more *domestic violence* (*z =* 2.92, *Pdunn =* 0.002, *d = 0*.63, CI [1.3, 5.6]), *abuse* (*z =* 2.74, *Pdunn =* 0.003, *d =* 0.41, CI [0.04, 3.14]), and *parental neglect* (*z =* 2.68, *Pdunn =* 0.004, *d =* 0.4, CI [0.96, 6.64]), while HACE students reported more *neighborhood insecurity* than ACCA-group (*z =* 5.87, *Pdunn˂*0.001, *d =* 1.7, CI [3.73, 6.08]).

### Objective Three: Emotional Competences Differences

#### ***Emotion Recognition***

**Fig. 4:**
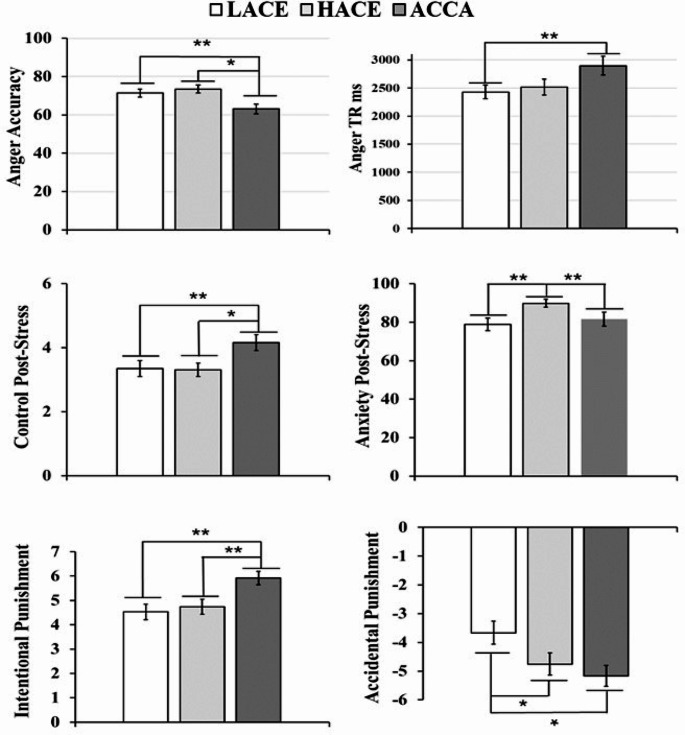
Comparison of Emotional Competences LACE, HACE, and ACCA Groups. *Note.* Comparison of emotional competences for recognizing (top), responding (middle), and empathizing to emotions (bottom) among LACE, HACE and ACCA. Significant differences have a *p-value* of **p˂0.01 and *p˂0.05

The analyses of variance were only significant for *anger perception. Childhood experience* significantly explained the accuracy of recognizing angry faces (χ^2^ (2, 149) = 8.49, *p =* 0.014). As is seen in Fig. 4, ACCA-adolescents were the less accurate (ACCA vs. LACE, *z* = -2.57, *Pdunn =* 0.017, *d=-*0.51, CI [-15.98, -0.65]; ACCA vs. HACE, *z=*-2.58, *Pdunn =* 0.002, *d =* 0.64, CI [-18.1, -2.72]). The time to recognize this emotion (latencies) was affected by *childhood experiences* (χ^2^ (2, 149) = 9.72, *p =* 0.008), with ACCA-adolescents being slower than LACE ones (*z* = 3.2, *Pdunn =* 0.002, *d =* 0.55, CI [-23.3, 958]). There were no significant differences in other emotions.

#### ***Social Stress Response***

The Kruskal-Wallis test showed that childhood experience explained control perception after social pressures (χ2(2, 149) = 7.77, *p* = 0.021). As depicted in Fig. 4, adolescents with ACCA history reported more control perception than LACE (z = 2.6, Pdunn = 0.009, d = 0.41, CI [0.11, 1.29]) and HACE teens (z = 2.35, Pdunn = 0.019, d = 0.41, CI [0.13, 1.3]). Finally, the Kruskal-Wallis test for STAI revealed that childhood experience had significant effects on post-stress anxiety (χ2(2, 149) = 14.36, *p* < 0.001). As is seen in Fig. 4, HACE-teens were more anxious than other groups (HACE vs. LACE z = 3.25, Pdunn = 0.001, d = 0.644, CI [3.83, 23.16]; HACE vs. ACCA z = 3.22, Pdunn = 0.001, d = 0.269, CI=[-4.94, 15.73]).

#### ***Empathy***

Repeated measurements ANOVAs for emotional concern and disconfirm did not produce significant differences in responses and time responses among pain situations. Repeated measurements ANOVA for *punishment* indicated that the *pain situation x group* significantly explained these answers (χ^2^ (4, 296) *=* 5.83, *p˂*0.001). In the intentional harm (Fig. 4), ACCA adolescents were clearly more punishers than LACE teens (*z =* 3.29, *Pdunn =* 0.001, *d =* 0.64, CI [-0.13, 2.24]). *Post-hoc* comparisons for accidental situations revealed that LACE adolescents gave more sanctions than the ACCA (*z =* 2.51, *Pdunn =* 0.012, *d=-*0.66, CI [0.16, 2.84]) and HACE groups (*z =* 2.1, *Pdunn =* 0.037, *d=*-0.38, CI [-0.15, 2.33]). Analyses of APS indicated that ACCA participants used more time to penalize the accidental damage than the HACE group (*z =* 2.27, *Pdunn =* 0.012, *d =* 0.62, CI [-3.5, -0.5]).

#### Objective Four: Behavioral Problems

**Fig. 5:**
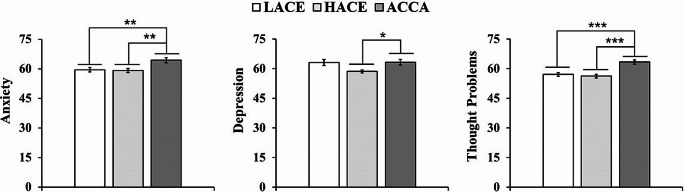
Comparison of parental reports of behavioral problems among LACE, HACE, and ACCA groups. *Note.* Comparison of parental reports of behavioral problems among LACE, HACE, and ACCA groups. The figure displays a parental report of problems in *anxiety*; *depression*; and *thought* problems. Significant differences correspond to a *p-*value of ***p˂0.001, **p˂0.01, *p˂0.05

Parental report of adolescents’ *behavioral problems* was associated with childhood experiences (χ^2^ (2, 158) = 11.24, *p* = 0.004); parents of adolescent war victims reported more *internalizing problems* than parents of the HACE group (*z* = 2.79, *Pdunn* = 0.003, *d* = 0.63, CI [0.92, 9.67]). The main differences were reported for anxiety and thought problems (Fig. 5). ACCA-teens were seen as more *anxious* (ACCA vs. HACE, *z =* 2.97, *Pdunn =* 0.001, *d =* 0.71, CI [1.77, 9.42]; ACCA vs. LACE, *z =* 2.97, *Pdunn =* 0.001, *d =* 0.75, CI [2.37, 10.24]). Finally, childhood experiences significantly explained thinking problems (*χ*^2^ (2, 150) = 8.01, *p*˂0.001) since the ACCA adolescent had the worst ratings (ACCA vs. LACE, *z =* 4.33, *Pdunn* ˂0.001, *d =* 0.814, CI [1.68, 10.14]; and ACCA vs. LACE, *z =* 4.39, *p˂*0.001, *d =* 0.98, CI [2.67, 10.87]).

#### Objective Five. Mediation Model

Seven models with mediational relationships were compared through structural equation modelling. As shown in Table S3 (supplementary material), Model 3 had the lowest AIC, BIC, and PNFI indicators and the highest CFI, thus considered the best-fitting one. In this model, ACCA exposure was taken as the predictor; the mediators were processing of anger expressions, sense of control after a social stressor, and degree of punishment for intentional harm; and the outcomes were all behavioral problems. On the other hand, Model 5 showed the worst fit indicators (CFI and PNFI); in this model, behavioral problems were used as mediators and emotional competencies as outcomes.

The supplementary material contains the tables corresponding to the mediational analysis to model 3. Total effects, total indirect effects, direct effects, indirect effects, residual covariances, and path coefficients are reported. The model showed that the total effects between ACCA and mental health and behavioral problems were not statistically significant (Table S6 and Table S7, supplementary material), indicating that all effects of ACCA on them were associated with changes in emotional competences.

Regarding indirect effects (Table S9, supplementary material), the model indicates the chain-mediating effect among ACCA, emotional competences, and mental health and behavioral problems. With moderate significance, that ACCA changes accuracy in recognizing angry faces, which modifies thought problems [Effect = -0.052, 95%, z = -1.976, C.I. (-0.126, -0.008), *p* = 0.048]. Additionally, with a weak significance, the model suggests the following mediated relationships: ACCA → time response for anger recognition → depression [Effect = -0.04, 95%, z = 1.600, C.I. (-0.085, -0.009), *p* = 0.11]; ACCA → control perception after social stress → social problems [Effect = -0.054, 95%, z = 1.827, C.I. (-0.138, -0.015), *p* = 0.068]; ACCA → punishment for intentional harms → aggressive behaviors [Effect = 0.055, 95%, z = 1.817, C.I. (0.007, 0.104), *p* = 0.069]; ACCA → punishment for intentional harms → thought problems [Effect = 0.048, 95%, z = 1.708, C.I. (-46.84, 0.118), *p* = 0.088]; and ACCA → punishment for intentional harms → social rule-breaking behaviors [Effect = 0.048, 95%, z = 1.665, C.I. (-0.002, 0.112), *p* = 0.096]. Regarding covariances (Table S10), no relationships were found between emotional competencies, but there were significant relationships among all behavioral problems.

**Fig. 6:**
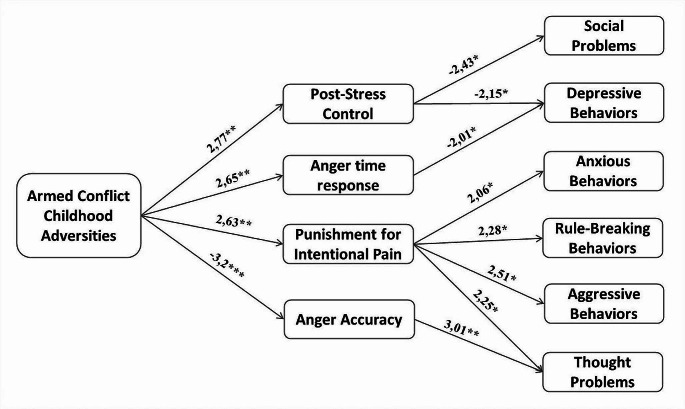
Proposed chain mediation model to explain the association between ACCA, post-stress control, anger time response, punishment for intentional pain, anger accuracy, social problems, depression, anxiety, rule-breaking behaviors, aggressive behaviors, and thought problem. *Note.* Z-values from path coefficients are shown, and significant differences correspond to a *p-*value of ***p˂0.001, **p˂0.01, *p˂0.05

Table S11 (supplementary material) shows the path coefficients, all paths were probe, but in the Fig. 6 only the significant paths were represented. The analysis of paths coefficients indicated: (1) ACCA increased the control perception after social pressures, which reduced the report of social problems and depressive behaviors; (2) longer response time to angry faces also led to lower depressive behaviors in adolescents who experienced ACCA; and (3) history of ACCA increased the punishment for intentional harms, which favored the reporting of mental health and behavioral problems as anxiety, rule-breaking, aggression, and thought problems. Finally, the analysis of path coefficients for the model where behavioral problems were the mediators indicated that the only inverse relationship between behavioral problems and emotional competencies occurred between thought problems and accuracy in recognizing angry faces, and exposure to ACCA did not directly predict any of the behavioral problems (Table S12).

## Discussion and Conclusions

### First Objective: Detect Cases of Exposure to ACCA in a Group of Adolescents from the Municipality of Soacha, Colombia

We found that many adolescents living in Soacha, Colombia have experienced a diversity of *armed conflict childhood adversities*, mainly forced displacement, and witnessed torture, combats, and murders. Furthermore, these findings confirm recent data indicating that this zone takes in a lot of families migrating from AC areas around the country (González-Montaño et al., 2019). Therefore, in the future, it will be essential to inquire deeply about the characteristics of these experiences and how they can affect the developmental trajectories of these teens. Moreover, these results highlight the importance of creating interventions within the Soacha communities targeted to reduce the vulnerability associated with ACCA.

### Second Objective: Comparing the Childhood Adversities Reported Among ACCA, LACE, and HACE Adolescents

We found that adolescents exposed to ACs experience domestic violence, abuse, and parental neglect more frequently, compared to adolescents who have not experienced these adversities in their childhood (Fig. 3). In the case of Colombia, the high accumulation of ACEs among adolescents exposed to ACs could be the result of highly unfavorable contexts among families living in regions where these conflicts occur; previous reports have highlighted the precarious situation faced by families in these regions, where extreme poverty, social exclusion, unemployment, and violence from armed groups abound, events that often increase rates of violence within families (Guerrero-Barón, 2011), which is consistent with our data. This accumulation of ACEs among children surviving ACs has been observed worldwide, where the fundamental rights of minors are often violated, causing frequent and intense suffering (Ainamani et al., 2020; Halevi et al., 2016; Hasanović, 2011).

The accumulation of adversities among adolescents growing up in ACs is not a minor issue; previous research has confirmed the harmful effect that this accumulation of ACEs has on the onset of psychosocial and health problems in adolescents (Hughes et al., 2017). Although not directly observed, this accumulation of ACEs in survivors of ACs may be related to the high rate of psychosocial problems reported in migrant youth living in Soacha, who present a higher risk of anxiety, depression, drug abuse, gang affiliation, and involvement in crimes than in other regions of Colombia (Castaño-Pérez et al., 2018). This hypothesis should be further investigated in the future to enrich community interventions conducted in regions where young people with AC experiences or high exposure to ACEs without ACCA are concentrated.

### Third Objective: Comparing Among ACCA, LACE, and HACE Groups the Emotional Competences to Recognize Facial Emotional Expressions, Respond to Social Stressors, and Empathize with Others’ Pain

Results from this study showed that the three emotional competences evaluated changed according to experiences of AC during childhood. Adolescents in the group ACCA had more problems recognizing the anger expressions of others (Fig. 4a and b). We could not find studies with similar results; on the contrary, researchers have reported that people exposed to terrorism, civil wars, and combat exhibit a bias to attend to more threatful cues, which makes them more hypervigilant, accurate, and fast in identifying fear and anger (Anaki et al., 2012; Ashley & Swick, 2019; Scrimin et al., 2009). A possible explanation for our result is that frequent and prolonged exposure to intense and uncontrollable threats, such as those associated with AC, could lead to an involuntary avoidance of these threatening stimuli, reducing sensitivity to hostile expressions. This hypothesis is supported by studies showing that severely abused adolescents with borderline and depressive disorders have significant difficulties recognizing anger (Bodenschatz et al., 2019; Seitz et al., 2021). Another possible reason for this decreased ability to recognize angry faces could be inconsistent exposure to facial expressions of emotions, limiting opportunities for training in recognizing these emotional displays. It has been documented that Colombian families living in conflict zones avoid showing signs of discomfort or confrontation because armed groups prohibit any challenge to their authority (Peltier-Bonneau & Szwarcberg, 2019). A final possible explanation for this finding could be related to the lack of ecological validity in the images used for this assessment of emotion perception competence, which were black-and-white photographs of American male and female models taken in 1976 (Kessels et al., 2013; Williams et al., 2022). Adolescents from rural contexts, where Armed Conflict occur, may have more difficulty identifying facial expressions in unfamiliar faces. Testing any of these hypotheses will require future studies using facial expression images with greater ecological validity to further explore the experience and neurophysiological response in adolescents who have experienced AC in their childhood.

After social pressure, youths exposed to ACCA tended to feel more control than other teenagers and less anxiety than teens who experienced HACE (Fig. 4c and d). Although we found no previous research on the response to social stress in adolescents exposed to ACCA, on the contrary, Pesonen et al. (2010) found that orphans in the context of war had a higher endocrine response during the TSST, which corresponds with other results showing that people raised in war contexts develop hormonal and physiological regulation problems, making it difficult for them to respond to daily challenges (Dajani et al., 2018; Shaheen et al., 2020). This evidence could indicate that the higher control perception and less anxiety during social pressures seen in teens affected by war are related to a blunted neuroendocrine reactivity for stressors, in the same way that other people experience extreme ACEs (Bunea et al., 2017; Trickett et al., 2014). Maybe the blunted stress response is associated with involuntary reorientation of attention towards less aversive stimuli, leading people exposed to severe childhood adversity to assess repetitive and uncontrollable threats as less dangerous (Milojevich et al., 2018; Roberts & Lopez-Duran, 2019). Further studies are needed to prove if the better control perception and lesser anxiety reported by the adolescents of this study who were victims of the Colombian war are consistent with changes in the neuroendocrine response to stress.

The moral responses during the pain empathy task were associated with the AC exposure. The adolescents with ACCA group tended to be more severe in assigning punishments (Fig. 4e), as they applied stronger punishments to intentional hurts but punished lightly if the damage was accidental. Particularly, these teenagers took longer to make their moral decisions. According to literature, a longer time in these responses is related to a more deliberate analysis of the harm situation, which may cause moral distancing during social scenarios requiring empathy (Wang et al., 2023). Following this line of argument, it is possible that the teens in the ACCA group base their punishment assignment for accidental hurts on a more rational and deliberative process. This hypothesis would be consistent with previous evidence that youths exposed to war tended to justify their moral actions with objectivist and vindictive ideas rather than with the mental and emotional states of others (Pasupathi et al., 2017; Posada & Wainryb, 2008). Moreover, a similar phenomenon has been consistently observed in combatants from different sides of the Colombian armed conflict, who tend to exhibit moral disengagement to justify their violent actions (Blanco et al., 2022; Villegas de Posada et al., 2018). Similarly, literature shows that adolescents with a history of extreme early adversity also developed moral disengagement, problems processing anger, and bullying behaviors (Gao et al., 2020; Wang et al., 2020). All these pieces of evidence support the idea that exposure to recurrent traumatic experiences during childhood would enhance moral thinking based on instrumental reasons and moral disengagement but not on empathic concern, justifying the application of severe punishments for hurts considered intentional (Gilède, 2012), which can support the extremely dehumanized actions commonly observed in men experiencing armed conflict (Baez et al., 2019; Çeri & Nasiroğlu, 2018). In the future, it would be interesting to assess theory of mind skills, establishing if there are differences in cognitive processing like perspective-taking and affective processing like emotional resonance.

### Fourth Objective: Comparing the Parental Report of Behavioral Problems Among ACCA, LACE, and HACE Teens

Parents of adolescents exposed to ACCA perceived their children had more thought problems, anxiety, and depression than parents of other adolescents (Fig. 5). Parental report of conduct problems has demonstrated to be highly consistent with the diagnosis of mental disorders (Rescorla et al., 2007), particularly in populations surviving armed conflicts (Hewitt-Ramírez et al., 2020; Montgomery, 2008). Therefore, our results seem to indicate that adolescents surviving AC have a higher risk of suffering from internalizing mental problems, which are consistent with most research supporting the idea that war-exposed youth are at increased risk of developing dysfunctional behaviors (Layne et al., 2010). In Colombia, there is consistent evidence showing that victims of the AC frequently develop internalization problems, which are correlated with suffering from chronic diseases (Hewitt-Ramírez et al., 2020). An explanation for this higher health risk is that the accumulation of childhood adversities causes an allostatic overload that sensitizes people to mental and physical problems (Bunea et al., 2017; Heim et al., 2000).

It is possible that the observed differences in parental perceptions about adolescent behavior are influenced by the same AC, so that parents who have lived in these threatening contexts may be biased to overestimate behavior problems in their children. Although there are no studies on these biases, there is evidence that parents who are survivors of AC are more likely to develop mental problems such as post-traumatic stress and anxiety disorders, disorders that favor the use of harsh parenting practices associated with a greater perception of behavior problems in their children (Back Nielsen et al., 2019; Bryant et al., 2018). The relationship between parents’ mental health, parenting practices, and children’s psychological adjustment in armed conflict contexts is a very important field of study to develop, since in this triple relationship it can cement more timely intervention strategies for families that flee and survive the AC.

### Fifth Objective: Exploring in a Chain Mediational Model What is the Role of Competences in Perceiving, Regulating and Empathizing with Others’ Emotions on the Report of Behavioral Problems in Adolescents that have Experienced Childhood Armed-Conflict

This is the first study showing evidence that early exposure to armed conflict adversities is related with changes in emotional competences to respond to social stressors, perceive emotions in others, and make moral decisions, and these changes can entail mental issues during adolescence (Fig. 6). Specifically, in the first path of this mediation model, we found that exposure to ACCA correlate with higher control perception after social stressors, which are related with lesser depressive symptoms and social problems. On one side, this makes sense as control perception facing social stress allows one to feel in control of the situation and have the opportunity to solve it. However, those who faced AC experiences and did not perceive a high control perception after social stressors showed more social problems perhaps due to that lack of control of the situation. On the other side, since this is the first result indicating the relationship between ACCA, better control during stress, and lower depression, it is hard to explain this tendency. Perhaps growing up in environments of AC provides some kind of insensitivity to mild or moderate social threats, or these ACCA experiences allow one to develop more confidence to manage social stressors (Luo et al., 2021). In both cases, this higher control perception protects against depressive behaviors. This hypothesis agrees with the inoculation of stress theory, which asserts that early threatful experiences may induce “inoculation” to future threats. Inoculated people become less sensitive to mild or moderate stressors, which favors managing social challenges (Ellis et al., 2017; Seitz et al., 2021). For instance, children living up to AC events could be inoculated in order to show more control across social stressors such as TSST, which makes others perceive them as more confident and less depressed. In the future, researchers must study deeper if, in some cases, early exposure to armed conflict events can be useful to control stressors and protect against depression.

In the second pathway, we found that longer time recognizing angry faces was related to lower reporting of depressive symptoms in adolescents with a history of AC; this result is novel but difficult to explain. As previously documented, adolescents exposed to extreme violence may be less adept at perceiving threatening cues, such as expressions of anger. This lower competence could make these adolescents less sensitive to social pressures that protect them from developing depressive behaviors. However, in the future, it will be essential to inquire necessary to investigate this possible mediation relationship in greater depth to determine if the result is consistent and what its implications. In the third pathway, AC experiences boosted the punishment for intentional aggressions, which enhanced three kinds of behavioral problems (thought problems, anxiety, rule-breaking, and aggressive behaviors). Previously, it was documented that the moral decisions seen in many survivors of armed conflicts fit a profile of moral disengagement, which could serve to reproduce acts of violence toward those who are considered deserving of these punishments (Blanco et al., 2022; Villegas de Posada et al., 2018). This is because morality mediated by prosocial and mutual care bonds blocks the expression of aggression towards others; therefore, disengagement from others’ needs and pains becomes a promoter of more impulsive antisocial behaviors (Kavussanu et al., 2015). However, these relationships and inferences must be proven in the future.

In the fourth pathway of the mediation model, we found that exposure to ACCA reduced the accuracy of identifying facial expressions of anger, which boosted the thought problems. The poor ability to identify anger can be related to the insensitivity to social threats described above; moreover, some evidence links emotion perception disturbances with disorganized thought (Yildirim et al., 2018). It is possible that anger identification imprecision indicates a neurodevelopmental adaptation in teens living up ACCA, which is also associated with a more impulsive and dysregulated behavior profile, which is consistent with items assessed in the CLCB Thought Problem Scale such as obsessive thoughts, self-harm, nervous twitching, compulsions, insomnia, strange behavior, and trouble sleeping.

In summary, we found enough evidence to assert that exposure to childhood armed-conflict adversities can generate variation in adolescent emotional competences, some of these can protect mental health, while others can lead to serious behavioral problems. The main explanation about the consequences of AC on mental development indicates that these threatful contexts disrupt economic, social, familial, and psychological life, leading to uncontrollable chronic stress and limited access to psychosocial resources to dampen it (Levy et al., 2019). Allostatic overload theory proposes that constant exposure to a toxic environment impacts brain plasticity, endocrine regulation, and immune response, leading to socioemotional dysfunctions (McEwen, 2016). The developmental effects of AC would depend on the moment of exposure and the specific social, cultural, and relational characteristics of each conflict (León-Rodríguez & Cardenas, 2021); thus, it is needed to compare our results with those of people reared in other regions at war. Therefore, this work can shed light on the specific consequences that exposure to ACs during childhood can leave, which can be valuable in constructing interventions aimed at promoting well-being in people who have grown up amid war.

### Policy Implications, Intervention, and Practice

Our findings could be relevant for designing interventions and public policy better targeted in the upcoming years. A structure of interventions based on the education of emotional competencies is suggested below, considering the context in which children and adolescents who are survivors of AC live and what their main needs are. Intervention programs should start with a strategy to conduct mass screenings to quantify the degree of exposure to ACEs and ACCA in children and adolescents through instruments such as the ACEQA. This screening can be carried out through public health or social protection systems, where doctors, nurses, psychologists, or social workers are trained to implement it. In a recent systematic review, the American College of Preventive Medicine has highlighted the importance of these screenings for the timely detection of psychosocial and health risks and for planning timely and effective, preventive and corrective interventions (Sherin et al., 2022). Based on these screenings, the population is segmented according to the level of detected risk, in order to prioritize the protection and care of groups with greater exposure to ACEs. Once the groups are segmented, it is suggested to carry out a family education plan based on healthy parenting practices and the development of emotional competencies within the family, as these interventions have shown high value in mitigating the negative effect of adversity in childhood. To carry out these strategies, it is advisable to take into account the detection of sociocultural and geographical barriers and facilitators specific to the contexts where the population of AC survivors is found. Government agencies can use health centers, rural schools, and community groups to implement these programs, which should involve social leaders recognized within the communities. More specific considerations on these family interventions in AC contexts appear in the reviews by De Jong (2020) and Jordans et al. (2016).

The implementation of structured interventions aimed at training social and emotional competencies in family members should be carried out through public schools or local community centers. A good example of a structured intervention strategy aimed at developing emotional competences in families and children who are victims of AC is the Pisotón program, which includes activities for children, caregivers, teachers, and school psychologists to train emotional expression and self-knowledge skills. The activities that took place in the classroom included games based on comics, role-playing games, and reflection spaces, while similar games were sent home to be played with parents and caregivers, who were required to send reflections on these experiences to the school teachers (Cosso et al., 2022). Another successful experience in training emotional competencies is the somatic emotional education program for teachers, which consists of seven body (somatic) training sessions using ancestral techniques such as Hatha Yoga, mindfulness, compassionate meditation, and breathing exercises. Each session was oriented by emotional themes from which competencies to perceive, understand, control, and use one’s and others’ emotions were trained (Castro J. et al., 2023). This program proved to be effective in improving emotional competencies in teachers, as well as achieving transfer of this learning to their students and their families. Additionally, other studies have shown that focusing emotional education programs on teachers has positive effects on the development of these skills in students and their families (Garcia-Vila et al., 2022; Harding et al., 2019).

A final recommendation for school interventions is that government agencies and educational policy managers design guidelines and programs to structure and strengthen psychosocial support in schools, particularly those that contain the most significant number of children and armed-conflict adolescent victims. These programs should facilitate better continuity and systematization of the work of school psychologists and counselors. In some contexts, psychologists carry out simple and short-term actions since they are not trained, or their contracts do not facilitate the monitoring and measuring of the impact of emotional education programs. In addition, school counselors tend to have many functions and tasks that prevent more meaningful and collaborative work with teachers, students, and parents (Cosso et al., 2022; Jordans et al., 2016).

### Limitations

One of the bigger limitations is that our sample size was small. Given many victims live in rural areas in conflict and forced displacement families face barriers to enrolling their children in schools, the contact with them is limited. Moreover, many people avoid participating in investigations due to the fear of being re-victimized, which is understandable considering that researchers are not always as sensible and empathetic as many victims need. Because of this, is very important for researchers to work together with the victims and what they need and to have the support of an ethical committee to develop protocols and strategies to not revictimize and even to promote well-being during and after the research project.

Another limitation is the lack of direct measures for mental health, familial relationships, and emotional regulation skills. This information would have allowed us to have a better picture of how emotional and social processes are developed in people growing up in AC environments. In addition, this study only used behavioral tasks to measure the AC marks, and other types of measures would be worthwhile for the research.

The limitations above show that, even though we found very interesting results, these cannot be generalized to the population. We cannot generalize our findings to other locations, as the way conflicts and adversities are experienced may vary across sociocultural contexts. However, they give us some interesting keys and highlight the importance and necessity of continuing to study the conflict and adversities consequences on children. We should continue learning about the victim’s history and context to make better research in this field being sensible to what they have lived and work together to create better comprehensions of the conflict and effective interventions highlighting socio-cultural approaches.

### Future Research Directions

Despite the limitations named above such as cultural variation, what it seems to be consistent is that chronic stress during childhood and the lack of protective networks, as occurs to a greater or lesser extent in all armed conflicts, affect the development of the body, emotional competencies, and social systems. All these impacts are reflected in a higher risk of bodily dysfunction (chronic diseases) and psychosocial dysfunction (mental illnesses). It would be important to observe if the mediation found in this study occurs in other conflict survivors, in order to adapt emotional education intervention protocols to the sociocultural contexts of the places where these conflicts occur. Future studies should get support from government and non-government organizations to include more participants, mainly those who inhabit the territories in conflict. Additionally, future research would have to investigate with greater precision what emotional and social skills are the most sensitive to the AC effects, which will allow us to unveil the paths that lead to violent reproduction and psychopathology (Betancourt et al., 2013). Translational research will allow a better comprehension of the epigenetic, genetic, neuroendocrine, and physiological mechanisms involved in the developmental impairments caused by ACCA, leading to the development of more efficient intervention strategies. For example, previous studies have found that ACs induce front-limbic disturbances by biassing affective assessment, facial perception, and relational inference (Joshi & O’Donnell, 2003). However, at present, the neurophysiological, neuroimage and neuroendocrine information about the psychobiological consequences of ACs on children and adolescents is limited.

On the transcultural research, previous works have indicated that an attentive, reflective, culturally sensitive, and trauma-aware healthcare workforce, alongside a supportive learning environment equipped with education, resources, and tools, can enhance the healthcare encounters of immigrants and refugees within the mental healthcare system (Wylie, 2018).

In conclusion, this article presents evidence indicating that changes in emotional competences mediate the presence of behavioral problems in adolescents who lived up to armed conflict childhood adversities. Educators and policymakers need to consider these results carefully to create better-targeted interventions aimed at improving the emotional competences and mental health of adolescent victims of armed conflicts.

## Electronic Supplementary Material

Below is the link to the electronic supplementary material.

Supplementary Material 1

## References

[CR1] Ainamani, H. E., Elbert, T., Olema, D. K., & Hecker, T. (2020). Gender differences in response to war-related trauma and posttraumatic stress disorder – a study among the Congolese refugees in Uganda. *Bmc Psychiatry*, *20*(1). 10.1186/s12888-019-2420-0. 10.1186/s12888-019-2420-0 PMC6954516 31924182

[CR2] Anaki, D., Brezniak, T., & Shalom, L. (2012). Faces in the face of death: Effects of exposure to life-threatening events and mortality salience on facial expression recognition in combat and noncombat military veterans. *Emotion*, *12*(4), 860–867. 10.1037/a0029415. 22866887 10.1037/a0029415

[CR3] Ashley, V., & Swick, D. (2019). Angry and fearful Face Conflict effects in post-traumatic stress disorder. *Frontiers in Psychology*, *10*, 136. 10.3389/fpsyg.2019.00136. 30804838 10.3389/fpsyg.2019.00136 PMC6370733

[CR4] Back Nielsen, M., Carlsson, J., Køster Rimvall, M., Petersen, J. H., & Norredam, M. (2019). Risk of childhood psychiatric disorders in children of refugee parents with post-traumatic stress disorder: A nationwide, register-based, cohort study. *The Lancet Public Health*, *4*(7), e353–e359. 10.1016/S2468-2667(19)30077-5. 31279418 10.1016/S2468-2667(19)30077-5

[CR81] Baez, S., Herrera, E., García, A. M., Huepe, D., Santamaría-García, H., & Ibáñez, A. (2018). Increased moral condemnation of accidental harm in institutionalized adolescents. *Scientific reports, 8*(1), 11609. https://doi.org/10.1038/s41598-018-29956-9 10.1038/s41598-018-29956-9 PMC6072742 30072749

[CR5] Baez, S., Santamaría-García, H., & Ibáñez, A. (2019). Disarming ex-combatants’ minds: Toward situated reintegration process in post-conflict Colombia. *Frontiers in Psychology*, *10*(JAN), 73. 10.3389/fpsyg.2019.00073. 30761041 10.3389/fpsyg.2019.00073 PMC6361777

[CR6] Betancourt, T. S., Borisova, I., Williams, T. P., Meyers-Ohki, S. E., Rubin-Smith, J. E., Annan, J., & Kohrt, B. A. (2013). Research Review: Psychosocial adjustment and mental health in former child soldiers - a systematic review of the literature and recommendations for future research. *Journal of Child Psychology and Psychiatry*, *54*(1), 17–36. 10.1111/j.1469-7610.2012.02620.x. 23061830 10.1111/j.1469-7610.2012.02620.x PMC4167714

[CR7] Biesanz, J. C., Falk, C. F., & Savalei, V. (2010). Assessing Mediational models: Testing and interval estimation for Indirect effects. *Multivariate Behavioral Research*, *45*(4), 661–701. 10.1080/00273171.2010.498292. 26735714 10.1080/00273171.2010.498292

[CR8] Blanco, A., Davies-Rubio, A., De la Corte, L., & Mirón, L. (2022). Violent extremism and Moral Disengagement: A study of Colombian Armed groups. *Journal of Interpersonal Violence*, *37*(1–2), 423–448. 10.1177/0886260520913643. 32228336 10.1177/0886260520913643

[CR9] Bodenschatz, C. M., Skopinceva, M., Ruß, T., & Suslow, T. (2019). Attentional bias and childhood maltreatment in clinical depression - an eye-tracking study. *Journal of Psychiatric Research*, *112*, 83–88. 10.1016/j.jpsychires.2019.02.025. 30870713 10.1016/j.jpsychires.2019.02.025

[CR10] Bryant, R. A., Edwards, B., Creamer, M., O’Donnell, M., Forbes, D., Felmingham, K. L., Silove, D., Steel, Z., Nickerson, A., McFarlane, A. C., Van Hooff, M., & Hadzi-Pavlovic, D. (2018). The effect of post-traumatic stress disorder on refugees’ parenting and their children’s mental health: A cohort study. *The Lancet Public Health*, *3*(5), e249–e258. 10.1016/S2468-2667(18)30051-3. 29731158 10.1016/S2468-2667(18)30051-3

[CR11] Bunea, I. M., Szentágotai-Tǎtar, A., & Miu, A. C. (2017). Early-life adversity and cortisol response to social stress: A meta-analysis. *Translational Psychiatry*, *7*(12). 10.1038/s41398-017-0032-3. 10.1038/s41398-017-0032-3 PMC5802499 29225338

[CR12] Campo-arias, A., Sanabria, A. R., Ospino, A., Guerra, V. M., Caama, B. H., & Magdalena, D. (2017). Original article multiple-victimisation due to armed conflict and emotional distress in the state of Magdalena. *6(3)*, *147-153*. 10.1016/j.rcpeng.2016.06.002. 10.1016/j.rcp.2016.06.005 28728798

[CR13] Cardona-Isaza, A., de Díaz-Posada, J., & enero-junio. (2021). L. E. (, Habilidades para la vida en jóvenes que han sido víctimas del conflicto armado en Colombia. *Revista Colombiana de Ciencias Sociales, 12*(1), pp. 76–94. 10.21501/22161201.3309.

[CR14] Castaño-Pérez, A., Sierra, G., Sánchez, D., Semenova, N., Salas, C., & Buitrago, C. (2018). Salud mental en víctimas de desplazamiento forzado por la violencia en Colombia. Editorial CES. ISBN 978-958-8674-59-9.

[CR15] Castro, J., León-Rodríguez (2023). Influencia de las técnicas somáticas en las prácticas pedagógicas y el bienestar emocional en contextos escolares. Instituto para la Investigación Educativa y el Desarrollo Pedagógico, IDEP. ISBN digital 978-628-7535-72-5.

[CR16] Çeri, V., & Nasiroğlu, S. (2018). The number of war-related traumatic events is associated with increased behavioural but not emotional problems among Syrian refugee children years after resettlement. *Revista De Psiquiatria Clinica*, *45*(4), 100–105. 10.1590/0101-60830000000167.

[CR17] Cerquera-Córdoba, A. M., Matajira-Camacho, Y. J., & Peña-Peña, A. J. (2020). Estrategias De Afrontamiento Y Nivel De Resiliencia Presentes en Adultos Jóvenes Víctimas Del Conflicto Armado Colombiano: Un Estudio Correlacional. *Psykhe (Santiago)*, *29*(2), 1–14. 10.7764/psykhe.29.2.1513.

[CR18] Cosso, J., de Vivo, A. R. R., Hein, S., Silvera, L. P. R., Ramirez-Varela, L., & Ponguta, L. A. (2022). Impact of a Social-emotional skills-Building Program (Pisotón) on Early Development of children in Colombia: A pilot effectiveness study. *International Journal of Educational Research*, *111*, 101898. 10.1016/J.IJER.2021.101898.

[CR19] Dajani, R., Hadfield, K., van Uum, S., Greff, M., & Panter-Brick, C. (2018). Hair cortisol concentrations in war-affected adolescents: A prospective intervention trial. *Psychoneuroendocrinology*, *89*, 138–146. 10.1016/j.psyneuen.2017.12.012. 29358120 10.1016/j.psyneuen.2017.12.012

[CR22] De Jong, J. T. V. M. (2020). Family interventions and armed conflict. *Cross-Cultural Family Research and Practice*, 437–475. 10.1016/B978-0-12-815493-9.00015-6.

[CR20] Decety, J., Michalska, K. J., & Kinzler, K. D. (2012). The contribution of emotion and cognition to moral sensitivity: a neurodevelopmental study. *Cerebral cortex (New York, N.Y.: 1991)*, *22*(1), 209–220. 10.1093/cercor/bhr111. 10.1093/cercor/bhr111 21616985

[CR21] Departamento Administrativo Nacional de Estadística [DANE] (2018). Censo Nacional de Población y Vivienda - CNPV 2018, proyección a 2022.

[CR23] DiGangi, J. A., Burkhouse, K. L., Aase, D. M., Babione, J. M., Schroth, C., Kennedy, A. E., & Phan, K. L. (2017). An electrocortical investigation of emotional face processing in military-related posttraumatic stress disorder. *Journal of Psychiatric Research*, *92*(2017), 132–138. 10.1016/j.jpsychires.2017.03.013. 28433950 10.1016/j.jpsychires.2017.03.013

[CR25] El Espectador. Soacha reporta reducción de embarazos en menores de edad y adolescentes (2022, April 18). *El Espectador*. Retrieved August 9, 2022, from https://www.elespectador.com/bogota/soacha-reporta-reduccion-considerable-de-embarazos-en-menores-de-edad-y-adolescentes/.

[CR24] Ellis, B. J., Bianchi, J. M., Griskevicius, V., & Frankenhuis, W. E. (2017). Beyond risk and protective factors: An adaptation-based Approach to Resilience. *Perspectives on Psychological Science*, *12*(4), 561–587. 10.1177/1745691617693054. 28679332 10.1177/1745691617693054

[CR26] Feldman, R., & Vengrober, A. (2011). Posttraumatic stress disorder in infants and young children exposed to war-related trauma. *Journal of the American Academy of Child and Adolescent Psychiatry*, *50*(7), 645–658. 10.1016/j.jaac.2011.03.001. 21703492 10.1016/j.jaac.2011.03.001

[CR27] Gao, L., Liu, J., Wang, W., Yang, J., Wang, P., & Wang, X. (2020). Moral disengagement and adolescents’ cyberbullying perpetration: Student-student relationship and gender as moderators. *Children and Youth Services Review*, *116*, 105119. 10.1016/J.CHILDYOUTH.2020.105119.

[CR28] Garcia-Vila, E., Sepúlveda-Ruiz, M. P., & Mayorga-Fernández, M. J. (2022). The emotional competences of the students of the teacher’s degrees in early childhood and primary education: An essential dimension in initial teacher training. *Revista Complutense De Educacion*, *33*(1), 119–130. 10.5209/RCED.73819.

[CR30] González-Montaño, J. N., Rodriguez-Molano, L. D., Tibacuy-Guzmán, S. A., Molina-Lopez;, K. J., & Escandon-Diaz, J. C. (2019). Analysis of youth unemployment in the Municipality of Soacha, causes and effects. *Perspectivas, 4*. Retrieved from https://revistas.uniminuto.edu/index.php/Pers/article/download/2075/1868/4830.

[CR31] Guerrero-Barón, H. (2011). *Afectación de la familia a causa del conflicto armado interno* (Vol. 6). Universidad Católica de Colombia. Retrieved from https://dialnet.unirioja.es/descarga/articulo/4459872.pdf.

[CR32] Gutierrez-Quintana, M. A. (2021). Validación por jueces de la adaptación de la Empathy Task para su aplicación en niños, niñas y adolescentes entre 7 y 14 años de Colombia. [Undergraduate Thesis, Universidad Antonio Nariño] Repositorio UAN. Retrieved from http://repositorio.uan.edu.co/handle/123456789/5150.

[CR33] Halevi, G., Djalovski, A., Vengrober, A., & Feldman, R. (2016). Risk and resilience trajectories in war-exposed children across the first decade of life. *Journal of Child Psychology and Psychiatry and Allied Disciplines*, *57*(10), 1183–1193. 10.1111/jcpp.12622. 27572904 10.1111/jcpp.12622

[CR34] Harding, S., Morris, R., Gunnell, D., Ford, T., Hollingworth, W., Tilling, K., Evans, R., Bell, S., Grey, J., Brockman, R., Campbell, R., Araya, R., Murphy, S., & Kidger, J. (2019). Is teachers’ mental health and wellbeing associated with students’ mental health and wellbeing? *Journal of Affective Disorders*, *242*, 180–187. 10.1016/j.jad.2018.08.080. 30189355 10.1016/j.jad.2018.08.080

[CR35] Hasanović, M. (2011). Psychological consequences of war-traumatized children and adolescents in Bosnia and Herzegovina. *Acta Medica Academica*, *40*(1), 45–66. Retrieved from https://www.ama.ba/index.php/ama/article/view/102.

[CR36] Heim, C., Newport, D. J., Heit, S., Graham, Y. P., Wilcox, M., Bonsall, R., Miller, A. H., & Nemeroff, C. B. (2000). Pituitary-adrenal and autonomic responses to stress in women after sexual and physical abuse in childhood. *Journal of the American Medical Association*, *284*(5), 592–597. 10.1001/jama.284.5.592. 10918705 10.1001/jama.284.5.592

[CR37] Hewitt-Ramírez, N., Juárez, F., Parada-Baños, A. J., Nuñez-Estupiñán, X., & Quintero-Barrera, L. (2020). Efficacy of a Primary Care Mental Health Program for victims of the Armed conflict in Colombia. *Peace and Conflict*, *26*(1), 62–77. 10.1037/PAC0000436.

[CR38] Hughes, K., Bellis, M. A., Hardcastle, K. A., Sethi, D., Butchart, A., Mikton, C., Jones, L., & Dunne, M. P. (2017). The effect of multiple adverse childhood experiences on health: A systematic review and meta-analysis. *The Lancet Public Health*, *2*(8), e356–e366. 10.1016/S2468-2667(17)30118-4. 29253477 10.1016/S2468-2667(17)30118-4

[CR39] JASP Team (2021). JASP (Version 0.16)[Computer software].

[CR40] Jordans, M. J. D., Pigott, H., & Tol, W. A. (2016). Interventions for children affected by Armed Conflict: A Systematic Review of Mental Health and Psychosocial support in low- and Middle-Income Countries. *Current Psychiatry Reports*, *18*(1), 1–15. 10.1007/S11920-015-0648-Z/TABLES/2. 26769198 10.1007/s11920-015-0648-z PMC4713453

[CR41] Joshi, P. T., & O’Donnell, D. A. (2003). Consequences of child exposure to war and terrorism. *Clinical Child and Family Psychology Review*, *6*(4), 275–292. 10.1023/B:CCFP.0000006294.88201.68. 14719639 10.1023/b:ccfp.0000006294.88201.68

[CR42] Kadir, A., Shenoda, S., & Goldhagen, J. (2019). Effects of armed conflict on child health and development: A systematic review. *PLOS ONE*, *14*(1), e0210071. 10.1371/journal.pone.0210071. 30650095 10.1371/journal.pone.0210071 PMC6334973

[CR43] Kavussanu, M., Stanger, N., & Ring, C. (2015). The effects of moral identity on moral emotion and antisocial behavior in sport. *Sport Exercise and Performance Psychology*, *4*(4), 268–279. 10.1037/spy0000040.

[CR44] Kessels, R. P. C., Montagne, B., Hendriks, A. W., et al. (2013). Assessment of perception of morphed facial expressions using the emotion Recognition Task: Normative data from healthy participants aged 8–75. *J Neuropsych*, *8*, 75–93. 10.1111/jnp.12009. 10.1111/jnp.12009 23409767

[CR45] Kithakye, M., Morris, A. S., Terranova, A. M., & Myers, S. S. (2010). The Kenyan political conflict and children’s adjustment. *Child Development*, *81*(4), 1114–1128. 10.1111/j.1467-8624.2010.01457.x. 20636685 10.1111/j.1467-8624.2010.01457.x

[CR46] Layne, C. M., Olsen, J. A., Baker, A., Legerski, J. P., Isakson, B., Pašalić, A., Duraković-Belko, E., Dapo, N., Ćampara, N., Arslanagić, B., Saltzman, W. R., & Pynoos, R. S. (2010). Unpacking trauma exposure risk factors and differential pathways of influence: Predicting postwar mental distress in Bosnian adolescents. *Child Development*, *81*(4), 1053–1076. 10.1111/j.1467-8624.2010.01454.x. 20636682 10.1111/j.1467-8624.2010.01454.x

[CR47] León-Rodríguez, D. A., & Cárdenas, F. (2021). Experiencias Adversas en la Niñez: Modificaciones Neuro-Estructurales, Neuro-Funcionales y Comportamentales. *PSYKHE 30*(2), 1–22. 10.7764/psykhe.2019.25213.

[CR48] Levy, J., Yirmiya, K., Goldstein, A., & Feldman, R. (2019). The neural basis of Empathy and Empathic Behavior in the Context of Chronic Trauma. *Frontiers in Psychiatry*, *10*. 10.3389/fpsyt.2019.00562. 10.3389/fpsyt.2019.00562 PMC6706815 31474883

[CR49] Ley 1448 de 2011 Por la cual se dictan medidas de atención, asistencia y reparación integral a las víctimas del conflicto armado interno y se dictan otras disposiciones. Junio 10 de 2011. Retrieved from https://www.funcionpublica.gov.co/eva/gestornormativo/norma.php?i=43043.

[CR50] Luo, J., Zhang, B., & Roberts, B. W. (2021). Sensitization or inoculation: Investigating the effects of early adversity on personality traits and stress experiences in adulthood. *Plos One*, *16*(4). 10.1371/JOURNAL.PONE.0248822. 10.1371/journal.pone.0248822 PMC8016298 33793582

[CR51] McEwen, B. S. (2016). In pursuit of resilience: Stress, epigenetics, and brain plasticity. *Annals of the New York Academy of Sciences*, *1373*(1), 56–64. 10.1111/nyas.13020. 26919273 10.1111/nyas.13020

[CR52] McLaughlin, K. A., Weissman, D., & Bitrán, D. (2019). Childhood adversity and neural development: A systematic review. *Annual Review of Developmental Psychology*, *1*, 277–312. 10.1146/annurev-devpsych-121318-084950. 32455344 10.1146/annurev-devpsych-121318-084950 PMC7243625

[CR53] Milojevich, H. M., Levine, L. J., Cathcart, E. J., & Quas, J. A. (2018). The role of maltreatment in the development of coping strategies. *Journal of Applied Developmental Psychology*, *54*, 23–32. 10.1016/j.appdev.2017.10.005. 32489226 10.1016/j.appdev.2017.10.005 PMC7266099

[CR54] Montgomery, E. (2008). Self- and Parent Assessment of Mental Health: Disagreement on Externalizing and Internalizing Behaviour in Young Refugees from the Middle East. 10.1177/1359104507086341. 10.1177/1359104507086341 18411865

[CR55] Østby, G., & Siri Aas Rustad & Andreas Forø Tollefsen. (2020). Children Affected by Armed Conflict, 1990–2019, *Conflict Trends, 6. Oslo: PRIO*. Retrieved from https://www.prio.org/publications/12884.

[CR56] Pasupathi, M., Wainryb, C., Bourne, S., & Posada, R. (2017). Narrative construction of morality in Adolescence among typically developing and violence-exposed youth. *Imagination Cognition and Personality*, *37*(2), 178–198. 10.1177/0276236617733826.

[CR57] Peltier-Bonneau, L., & Szwarcberg, M. (2019). Transformación De las emociones en las víctimas del conflicto armado para la reconciliación en Colombia. *Desafíos*, *31*(2), 197. https://doi.org/10. 10.12804/revistas.urosario.edu.co/desafios/a.7283.

[CR58] Pesonen, A. K., Räikkönen, K., Feldt, K., Heinonen, K., Osmond, C., Phillips, D. I., Barker, D. J., Eriksson, J. G., & Kajantie, E. (2010). Childhood separation experience predicts HPA axis hormonal responses in late adulthood: A natural experiment of World War II. *Psychoneuroendocrinology*, *35*(5), 758–767. 10.1016/j.psyneuen.2009.10.017. 19963324 10.1016/j.psyneuen.2009.10.017

[CR59] Pike, N. A., Poulsen, M. K., & Woo, M. A. (2017). Validity of the Montreal Cognitive Assessment Screener in adolescents and Young adults with and without congenital heart disease. *Nursing Research*, *66*(3), 222. 10.1097/NNR.0000000000000192. 28448372 10.1097/NNR.0000000000000192 PMC5408464

[CR61] Posada, R., & Wainryb, C. (2008). Moral development in a violent society: Colombian children’s judgments in the context of survival and revenge. *Child Development*, *79*(4), 882–898. 10.1111/j.1467-8624.2008.01165.x. 18717896 10.1111/j.1467-8624.2008.01165.x

[CR29] Posada Gilède, R. Experiencias, De Violencia Y Razonamiento Moral En, & Un Contexto De Venganza. (2012). *Revista Colombiana de Psicología*, *21*(2), 197–212.Retrieved from https://revistas.unal.edu.co/index.php/psicologia/article/view/28109.

[CR60] Registro Único de Victimas (2022, March 31). *Reporte general* Unidad para la atención y reparación integra a las víctimas. https://www.unidadvictimas.gov.co/es/registro-unico-de-victimas-ruv/37394.

[CR62] Rescorla, L., Achenbach, T., Ivanova, M. Y., Dumenci, L., Almqvist, F., Bilenberg, N., Bird, H., Chen, W., Dobrean, A., Döpfner, M., Erol, N., Fombonne, E., Fonseca, A., Frigerio, A., Grietens, H., Hannesdottir, H., Kanbayashi, Y., Lambert, M., Larsson, B., & Verhulst, F. (2007). Behavioral and Emotional Problems Reported by Parents of Children Ages 6 to 16 in 31 Societies. 10.1177/10634266070150030101.

[CR63] Roberts, A. G., & Lopez-Duran, N. L. (2019). Developmental influences on stress response systems: Implications for psychopathology vulnerability in adolescence. *Comprehensive Psychiatry*, *88*, 9–21. 10.1016/j.comppsych.2018.10.008. 30466015 10.1016/j.comppsych.2018.10.008

[CR64] Roupetz, S., Bartels, S. A., Michael, S., Najjarnejad, N., Anderson, K., & Davison, C. (2020). Displacement and emotional well-being among married and unmarried Syrian adolescent girls in Lebanon: An analysis of narratives. *International Journal of Environmental Research and Public Health*, *17*(12), 1–22. 10.3390/ijerph17124543. 10.3390/ijerph17124543 PMC7345669 32599758

[CR65] Save the Children (2019). *Stop the War on Children: Protecting Children in 21st Century Conflict* Save the Children. https://www.savethechildren.org/content/dam/usa/reports/ed-cp/stop-the-war-on-children-2019.

[CR66] Scrimin, S., Moscardino, U., Capello, F., Altoè, G., & Axia, G. (2009). Recognition of facial expressions of mixed emotions in School-Age Children exposed to Terrorism. *Developmental Psychology*, *45*(5), 1341–1352. 10.1037/a0016689. 19702396 10.1037/a0016689

[CR68] Seitz, K. I., Leitenstorfer, J., Krauch, M., Hillmann, K., Boll, S., Ueltzhoeffer, K., Neukel, C., Kleindienst, N., Herpertz, S. C., & Bertsch, K. (2021). An eye-tracking study of interpersonal threat sensitivity and adverse childhood experiences in borderline personality disorder. *Borderline Personality Disorder and Emotion Dysregulation*, *8*(1). 10.1186/s40479-020-00141-7. 10.1186/s40479-020-00141-7 PMC7784013 33397512

[CR67] Shaheen, M., Schindler, L., Saar-Ashkenazy, R., Bani Odeh, K., Soreq, H., Friedman, A., & Kirschbaum, C. (2020). Victims of war—psychoendocrine evidence for the impact of traumatic stress on psychological well-being of adolescents growing up during the Israeli–Palestinian conflict. *Psychophysiology*, *57*(1), e13271. 10.1111/PSYP.13271. 30101980 10.1111/psyp.13271

[CR69] Sherin, K. M., Stillerman, A. J., Chandrasekar, L., Went, N. S., & Niebuhr, D. W. (2022). Recommendations for Population-based applications of the adverse childhood experiences Study: Position Statement by the American College of Preventive Medicine. *AJPM Focus*, *1*(2), 100039. 10.1016/J.FOCUS.2022.100039. 37791246 10.1016/j.focus.2022.100039 PMC10546534

[CR70] Slone, M., & Mann, S. (2016). Effects of war, terrorism and armed conflict on young children: A systematic review. *Child Psychiatry and Human Development*, *47*(6), 950–965. 10.1007/s10578-016-0626-7. 26781095 10.1007/s10578-016-0626-7

[CR72] Tobón, C., Aguirre-Acevedo, D. C., Velilla, L., Duque, J., Ramos, C. P., & Pineda, D. (2016). Perfil psiquiátrico, cognitivo y de reconocimiento de características emocionales de un grupo de excombatientes de Los grupos armados ilegales en Colombia. *Revista Colombiana De Psiquiatria*, *45*(1), 28–36. 10.1016/j.rcp.2015.07.004. 26896402 10.1016/j.rcp.2015.07.004

[CR71] Trickett, P. K., Gordis, E., Peckins, M. K., & Susman, E. J. (2014). Stress reactivity in Maltreated and Comparison Male and Female Young adolescents. *Child Maltreatment*, *19*(1), 27–37. 10.1177/1077559513520466. 24482544 10.1177/1077559513520466

[CR73] Umiltà, M. A., Wood, R., Loffredo, F., Ravera, R., & Gallese, V. (2013). Impact of civil war on emotion recognition: The denial of sadness in Sierra Leone. *Frontiers in Psychology*, *4*, 523. 10.3389/fpsyg.2013.00523. 24027541 10.3389/fpsyg.2013.00523 PMC3760028

[CR75] Villegas de Posada, M. C., Flórez, J., & Espinel, N. (2018). Moral Disengagement mechanisms and Armed Violence. A comparative study of paramilitaries and guerrillas in Colombia. *Revista Colombiana De Psicología*, *27*(1), 55–69. 10.15446/rcp.v27n1.62191.

[CR74] Viola, T., Wendt, Wearick-Silva, L., Eduardo, Tractenberg, S. Gantes, & Grassi-Oliveira, R. (2014). Translation and adaptation of the trier social stress test for children into Portuguese language. *Temas em Psicologia*, *22*(3), 655–662. 10.9788/TP2014.3-10.

[CR77] Wang, X., Zhao, F., Yang, J., Gao, L., Li, B., Lei, L., & Wang, P. (2020). Childhood maltreatment and bullying perpetration among Chinese adolescents: A Moderated Mediation Model of Moral disengagement and trait anger. *Child Abuse & Neglect*, *106*. 10.1016/J.CHIABU.2020.104507. 10.1016/j.chiabu.2020.104507 32361515

[CR76] Wang, H., Ao, L., Gao, Y., Liu, Y., & Zhang, X. (2023). Empathy for pain in individuals influenced by moral identity: Evidence from an ERP study. *Physiology & Behavior*, *266*, 114202. 10.1016/j.physbeh.2023.114202. 37084861 10.1016/j.physbeh.2023.114202

[CR78] Williams, T. F., Vehabovic, N., & Simms, L. J. (2022). Developing and validating a facial emotion Recognition Task with graded intensity. *Assessment*, *10731911211068084*. 10.1177/10731911211068084. Advance online publication. 10.1177/10731911211068084 34991368

[CR79] Wylie, L., Van Meyel, R., Harder, H., Sukhera, J., Luc, C., Ganjavi, H., Elfakhani, M., & Wardrop, N. (2018). Assessing trauma in a transcultural context: Challenges in mental health care with immigrants and refugees. *Public Health Reviews*, *39*, 22. 10.1186/s40985-018-0102-y. 30151315 10.1186/s40985-018-0102-y PMC6103972

[CR80] Yildirim, E., Yalinçetin, B., Sevilmiş, Ş., Kutay, Ö., & Alptekin, K. (2018). Is there any relation between impaired emotion perception and thought disorder in Schizophrenia? *Noro Psikiyatri Arsivi*, *55*(2), 118–122. 10.5152/npa.2017.19277. 30057451 10.5152/npa.2017.19277 PMC6060655

