# Supplementary material for: Emotional Competences in Adolescents Exposed to Colombian Armed Conflict During Their Childhood
Source: J Child Adolesc Trauma. 2024 Jul 30;17(4):1133–50. doi: 10.1007/s40653-024-00647-0 (PMC11646258; doi:10.1007/s40653-024-00647-0)
Supplement: Supplementary file 1 — Supplementary Material 1 [file 40653_2024_647_MOESM1_ESM.docx]

**Supplementary Material**

**Descriptive statistics**

Each table shows mean, standard error of mean (SEM), median, standard deviation (SD), normality test, and homogeneity test. Normality and homogeneity were valuated with Shapiro-Wilk and Levene tests respectively.

**Table S1**

*Descriptive results of emotion recognition*

| **Emotions** | **Mean** | **SEM** | **Median** | **SD** | **Shapiro-Wilk** | **P-value S-W** | **Levene's test** | **P-value L** |
| --- | --- | --- | --- | --- | --- | --- | --- | --- |
| Happiness | 96.562 | 0.736 | 100 | 9.344 | 0.4 | < .001 | 0.51 | 0.601 |
| Surprise | 89.752 | 1.021 | 100 | 12.952 | 0.77 | < .001 | 0.114 | 0.892 |
| Disgust | 53.339 | 1.72 | 62.5 | 21.819 | 0.925 | < .001 | 0.003 | 0.997 |
| Anger | 69.953 | 1.305 | 75 | 16.558 | 0.94 | < .001 | 3.563 | 0.031 |
| Fear | 49.025 | 1.763 | 50 | 22.365 | 0.966 | < .001 | 1.31 | 0.273 |
| Sadness | 58.385 | 1.897 | 62.5 | 24.068 | 0.966 | < .001 | 0.43 | 0.651 |
| TR Happiness | 1326.412 | 54.283 | 1215 | 688.779 | 0.85 | < .001 | 0.532 | 0.589 |
| TR Disgust | 2361.507 | 88.767 | 2129 | 1126.333 | 0.811 | < .001 | 0.247 | 0.781 |
| TR Anger | 2587.13 | 82.192 | 2344 | 1042.898 | 0.872 | < .001 | 0.319 | 0.727 |
| TR Fear | 2301.342 | 82.762 | 2125 | 1050.129 | 0.739 | < .001 | 4.259 | 0.016 |
| TR Surprise | 1892.595 | 67.656 | 1710.625 | 858.46 | 0.734 | < .001 | 1.307 | 0.274 |
| TR Sadness | 2515.583 | 78.846 | 2342 | 1000.447 | 0.881 | < .001 | 2.307 | 0.103 |

**Table S2**

*Descriptive results of empathy for pain*

| **EPT** | **Mean** | **SEM** | **Median** | **SD** | **Shapiro-Wilk** | **P-value S-W** | **Levene's test** | **P-value L** |
| --- | --- | --- | --- | --- | --- | --- | --- | --- |
| IPS-Empathic | 3.016 | 0.289 | 3.5 | 3.655 | 0.949 | < .001 | 1.986 | 0.084 |
| IPS-Discomfort | 4.321 | 0.213 | 4.5 | 2.699 | 0.965 | < .001 | 0.497 | 0.778 |
| IPS-Correctness | 5.54 | 0.178 | 6.042 | 2.254 | 0.925 | < .001 | 2.843 | 0.017 |
| IPS-Punishment | 5.048 | 0.198 | 5.5 | 2.509 | 0.875 | < .001 | 1.671 | 0.145 |
| APS-Empathic | -1.593 | 0.268 | -1.5 | 3.393 | 0.98 | 0.018 | 2.352 | 0.043 |
| APS-Discomfort | -1.987 | 0.248 | -1.5 | 3.137 | 0.981 | 0.027 | 1.054 | 0.388 |
| APS-Correctness | -4.159 | 0.22 | -4 | 2.782 | 0.958 | < .001 | 0.458 | 0.807 |
| APS-Punishment | -4.262 | 0.236 | -4.358 | 2.985 | 0.956 | < .001 | 1.55 | 0.178 |
| TR IPS-Empathic | 2435.592 | 124.531 | 2027.625 | 1575.202 | 0.808 | < .001 | 2.021 | 0.079 |
| TR IPS-Discomfort | 2497.875 | 134.077 | 1952.25 | 1695.961 | 0.866 | < .001 | 3.436 | 0.006 |
| TR IPS-Correctness | 2221.854 | 110.983 | 1962.15 | 1403.835 | 0.863 | < .001 | 1.2 | 0.312 |
| TR IPS-Punishment | 2618.667 | 151.502 | 2149.5 | 1916.37 | 0.847 | < .001 | 2.069 | 0.072 |
| TR APS-Empathic | 2776.644 | 150.331 | 2382.875 | 1901.555 | 0.749 | < .001 | 1.373 | 0.238 |
| TR APS-Discomfort | 2764.769 | 137.015 | 2299.8 | 1733.117 | 0.895 | < .001 | 1.389 | 0.231 |
| TR APS-Correctness | 2893.48 | 171.829 | 2528.875 | 2173.479 | 0.795 | < .001 | 1.14 | 0.342 |
| TR APS-Punishment | 3009.947 | 166.621 | 2522.525 | 2107.611 | 0.842 | < .001 | 2.257 | 0.051 |

**Table S3**

*Descriptive results of behavior results*

| **Behavior Problems** | **Mean** | **SEM** | **Median** | **SD** | **Shapiro-Wilk** | **P-value S-W** | **Levene's test** | **P-value L** |
| --- | --- | --- | --- | --- | --- | --- | --- | --- |
| Anxiety | 60.677 | 0.656 | 60 | 8.321 | 0.936 | < .001 | 3.19 | 0.009 |
| Depression | 61.503 | 0.739 | 59 | 9.372 | 0.898 | < .001 | 4.156 | 0.001 |
| Somatic | 64.938 | 0.71 | 64 | 9.003 | 0.972 | 0.002 | 1.096 | 0.365 |
| Social | 60.168 | 0.602 | 60 | 7.64 | 0.942 | < .001 | 0.929 | 0.464 |
| Thought | 58.491 | 0.604 | 57 | 7.663 | 0.878 | < .001 | 0.401 | 0.847 |
| Inattention | 56.093 | 0.462 | 56 | 5.861 | 0.832 | < .001 | 1.274 | 0.278 |
| Rule-Breaking | 55.478 | 0.375 | 54 | 4.754 | 0.858 | < .001 | 1.449 | 0.21 |
| Aggressivity | 59.391 | 0.563 | 58 | 7.148 | 0.931 | < .001 | 2.208 | 0.056 |
| Behavior Problems | 59.335 | 0.707 | 60 | 8.974 | 0.984 | 0.069 | 0.98 | 0.432 |

**Models Comparison**

Six hypothetical models were designed as described in Table X. These models were designed based on the results of previous phases. Not all responses in the emotional competence tasks were included as mediators or outcomes because this large number of measures saturated the processing capacity of the statistical program.

Table Z shows the fit indices for each of the models. The models are presented in descending order from the best to the worst fit. According to the fit indices, Model 3 showed the lowest AIC, BIC, and PNFI values and the highest CFI, so it is considered the best fit. Model 5, in which behavioral problems are taken as mediators, shows the poorest fit according to the AIC, BIC, and CFI indices. Therefore, it is more prudent to affirm that emotional competencies may lead to behavioral problems, and it is very difficult to maintain the hypothesis that behavioral problems cause disturbances in emotional functioning.

**Table S4**

*Models to Mediation Analysis*

| **Model** | **Predictors** | **Mediators** | **Outcomes** |
| --- | --- | --- | --- |
| 1 | ACCA | Anger TR + Anger + Post Control + Stai post + APS-Empathic + IPS punishment + APS punishment | Internalizing problems and externalizing problems |
| 2 | ACCA | Anger TR + Anger + Post Control + Stai post + APS-Empathic + IPS punishment + APS punishment | Anxiety, somatic, depression, social, inattention, rule-breaking, thought and aggressive problems |
| 3 | ACCA | Anger TR + Anger + Post Control + IPS punishment | Anxiety, somatic, depression, social, inattention, rule-breaking, thought and aggressive problems |
| 4 | ACCA | Anger TR + Anger + Post Control + Stai post + APS-Empathic + IPS punishment + APS punishment | Anxiety, depression, rule-breaking, thought and aggressive problems |
| 5 | ACCA | Anxiety, somatic, depression, social, inattention, rule-breaking, thought and aggressive problems | Anxiety, somatic, depression, social, inattention, rule-breaking, thought and aggressive problems |
| 6 | ACCA + Sex | Anger TR + Anger + Post Control + Stai post + APS-Empathic + IPS punishment + APS punishment | Anxiety, somatic, depression, social, inattention, rule-breaking, thought and aggressive problems |
| 7 | ACCA | Anger TR + Anger + Post Control + Stai post + IPS punishment + APS punishment | Index of Beauvoir Problem |

**Table S5**

*Fit indeces*

|  |  |  | **Baseline test** | | | **Difference test** | | |  |  |
| --- | --- | --- | --- | --- | --- | --- | --- | --- | --- | --- |
|  | **AIC** | **BIC** | **χ²** | **df** | **p** | **Δχ²** | **Δdf** | **p** | **CFI** | **PNFI** |
| Model 3 | 10029.2 | 10189.4 | 64.781 | 21 | < .001 |  |  |  | 1.000 | 0 |
| Model 4 | 11728.7 | 11929.0 | 29.459 | 15 | 0.014 | 13.368 | 10 | 0.204 | 0.995 | 0.179 |
| Model 2 | 15519.5 | 15883.1 | 0 | 0 | 1.000 | 16.091 | 5 | 0.007 | 0.982 | 0.138 |
| Model 6 | 15522.9 | 15929.6 | 13.368 | 10 | 0.204 | 0.17 | 0 |  | 0.985 | 0.123 |
| Model 1 | 10864.7 | 10994.1 | 408.599 | 21 | < .001 | 35.152 | 6 | < .001 | 0.878 | 0.392 |
| Model 5 | 16319.8 | 16664.9 | 29.629 | 15 | 0.013 | 343.817 | 0 |  | 0.441 | 0.098 |
| Model 7 | 9794.0 | 9889.5 | 64.781 | 21 | < .001 | -343.81 | 0 |  | 0.845 | 0.465 |

*Note.* Comparative Fit Index (CFI), Parsimony Normed Fit Index (PNFI)

**Mediation Analysis**

**Table S6**

*Total Effects*

|  | | | | | | | **95% Confidence Interval** | |
| --- | --- | --- | --- | --- | --- | --- | --- | --- |
|  |  |  | **Estimate** | **Std. Error** | **z-value** | **p** | **Lower** | **Upper** |
| ACCA | → | Anxiety | -0.02 | 0.079 | -0.251 | 0.802 | -0.115 | 0.126 |
| ACCA | → | Depression | 0.037 | 0.079 | 0.47 | 0.639 | -0.125 | 0.16 |
| ACCA | → | Thought P. | 0.076 | 0.079 | 0.958 | 0.338 | -0.069 | 0.22 |
| ACCA | → | Rule-Breaking | 0.126 | 0.078 | 1.611 | 0.107 | -0.051 | 0.238 |
| ACCA | → | Somatic P. | 0.051 | 0.079 | 0.644 | 0.52 | -0.114 | 0.182 |
| ACCA | → | Social P. | 0.131 | 0.078 | 1.672 | 0.094 | -0.026 | 0.301 |
| ACCA | → | Inattention | 0.133 | 0.078 | 1.702 | 0.089 | 0.002 | 0.281 |
| ACCA | → | Aggressivity | 0.093 | 0.079 | 1.187 | 0.235 | -0.051 | 0.215 |
| *Note.*  Delta method standard errors, normal theory confidence intervals, ML estimator. | | | | | | | | |

**Table S7**

*Total Indirect Effects*

|  | | | | | | | **95% Confidence Interval** | |
| --- | --- | --- | --- | --- | --- | --- | --- | --- |
|  |  |  | **Estimate** | **Std. Error** | **z-value** | **p** | **Lower** | **Upper** |
| ACCA | → | Anxiety | -0.027 | 0.06 | -0.451 | 0.652 | -0.178 | 0.168 |
| ACCA | → | Depression | -0.066 | 0.059 | -1.130 | 0.258 | -0.271 | 0.067 |
| ACCA | → | Thought P. | -0.056 | 0.06 | -0.941 | 0.347 | -0.192 | 0.138 |
| ACCA | → | Rule-Breaking | 0.016 | 0.058 | 0.282 | 0.778 | -0.206 | 0.117 |
| ACCA | → | Somatic P. | 0.051 | 0.06 | 0.854 | 0.393 | -0.12 | 0.183 |
| ACCA | → | Social P. | -0.044 | 0.06 | -0.745 | 0.456 | -0.19 | 0.051 |
| ACCA | → | Inattention | -0.03 | 0.057 | -0.532 | 0.595 | -0.275 | 0.137 |
| ACCA | → | Aggressivity | 0.007 | 0.059 | 0.11 | 0.912 | -0.192 | 0.161 |
| *Note.*  Delta method standard errors, normal theory confidence intervals, ML estimator. | | | | | | | | |

**Table S8**

*Direct Effects*

|  | | | | | | | **95% Confidence Interval** | |
| --- | --- | --- | --- | --- | --- | --- | --- | --- |
|  |  |  | **Estimate** | **Std. Error** | **z-value** | **p** | **Lower** | **Upper** |
| ACCA | → | Anxiety | 0.007 | 0.082 | 0.087 | 0.931 | -0.202 | 0.233 |
| ACCA | → | Depression | 0.103 | 0.084 | 1.226 | 0.22 | -0.099 | 0.298 |
| ACCA | → | Thought P. | 0.132 | 0.082 | 1.618 | 0.106 | -0.086 | 0.337 |
| ACCA | → | Rule-Breaking | 0.11 | 0.085 | 1.298 | 0.194 | -0.124 | 0.336 |
| ACCA | → | Somatic P. | -58.82 | 0.081 | -0.007 | 0.995 | -0.171 | 0.192 |
| ACCA | → | Social P. | 0.162 | 0.084 | 1.932 | 0.053 | -0.002 | 0.327 |
| ACCA | → | Inattention | 0.164 | 0.086 | 1.904 | 0.057 | -0.043 | 0.347 |
| ACCA | → | Aggressivity | 0.087 | 0.083 | 1.048 | 0.295 | -0.102 | 0.225 |
| *Note.*  Delta method standard errors, normal theory confidence intervals, ML estimator. | | | | | | | | |

**Table S9**

*Indirect Effects*

|  | | | | | | | | | **95% Confidence Interval** | |
| --- | --- | --- | --- | --- | --- | --- | --- | --- | --- | --- |
|  |  |  |  |  | **Estimate** | **Std. Error** | **z-value** | **P** | **Lower** | **Upper** |
| ACCA | → | Anger | → | Anxiety | 0.002 | 0.02 | 0.092 | 0.927 | -0.037 | 0.04 |
| ACCA | → | TR Anger | → | Anxiety | -0.016 | 0.017 | -0.909 | 0.363 | -0.05 | 0.018 |
| ACCA | → | Post Control | → | Anxiety | -0.031 | 0.02 | -1.540 | 0.124 | -0.071 | 0.009 |
| ACCA | → | IPS-Punishment | → | Anxiety | 0.036 | 0.021 | 1.713 | 0.087 | -0.005 | 0.078 |
| ACCA | → | Anger | → | Depression | -39.41 | 0.02 | -0.018 | 0.986 | -0.039 | 0.038 |
| ACCA | → | TR Anger | → | Depression | -0.028 | 0.019 | -1.423 | 0.155 | -0.066 | 0.01 |
| ACCA | → | Post Control | → | Depression | -0.034 | 0.021 | -1.629 | 0.103 | -0.075 | 0.007 |
| ACCA | → | IPS-Punishment | → | Depression | 0.013 | 0.017 | 0.745 | 0.456 | -0.021 | 0.046 |
| ACCA | → | Anger | → | Thought P. | -0.057 | 0.026 | -2.193 | 0.028* | -0.109 | -0.006 |
| ACCA | → | TR Anger | → | Thought P. | -0.017 | 0.017 | -1.013 | 0.311 | -0.051 | 0.016 |
| ACCA | → | Post Control | → | Thought P. | -0.017 | 0.018 | -0.954 | 0.34 | -0.051 | 0.018 |
| ACCA | → | IPS-Punishment | → | Thought P. | 0.04 | 0.022 | 1.822 | 0.068 | -0.003 | 0.082 |
| ACCA | → | Anger | → | Rule-Breaking | 0.012 | 0.02 | 0.588 | 0.557 | -0.027 | 0.051 |
| ACCA | → | TR Anger | → | Rule-Breaking | -0.019 | 0.018 | -1.071 | 0.284 | -0.054 | 0.016 |
| ACCA | → | Post Control | → | Rule-Breaking | -0.017 | 0.018 | -0.972 | 0.331 | -0.053 | 0.018 |
| ACCA | → | IPS-Punishment | → | Rule-Breaking | 0.037 | 0.021 | 1.722 | 0.085 | -0.005 | 0.078 |
| ACCA | → | Anger | → | Aggressivity | -0.021 | 0.021 | -1.025 | 0.306 | -0.062 | 0.019 |
| ACCA | → | TR Anger | → | Aggressivity | -0.003 | 0.016 | -0.167 | 0.867 | -0.035 | 0.03 |
| ACCA | → | Post Control | → | Aggressivity | -0.021 | 0.019 | -1.108 | 0.268 | -0.057 | 0.016 |
| ACCA | → | IPS-Punishment | → | Aggressivity | 0.026 | 0.019 | 1.381 | 0.167 | -0.011 | 0.064 |
| ACCA | → | Anger | → | Somatic P. | 0.036 | 0.023 | 1.592 | 0.111 | -0.008 | 0.08 |
| ACCA | → | TR Anger | → | Somatic P. | -0.026 | 0.019 | -1.354 | 0.176 | -0.063 | 0.012 |
| ACCA | → | Post Control | → | Somatic P. | -0.023 | 0.019 | -1.205 | 0.228 | -0.059 | 0.014 |
| ACCA | → | IPS-Punishment | → | Somatic P. | 0.02 | 0.018 | 1.104 | 0.27 | -0.015 | 0.055 |
| ACCA | → | Anger | → | Social P. | -0.006 | 0.02 | -0.326 | 0.745 | -0.045 | 0.032 |
| ACCA | → | TR Anger | → | Social P. | -0.01 | 0.017 | -0.619 | 0.536 | -0.043 | 0.023 |
| ACCA | → | Post Control | → | Social P. | -0.033 | 0.021 | -1.598 | 0.11 | -0.074 | 0.007 |
| ACCA | → | IPS-Punishment | → | Social P. | 0.019 | 0.018 | 1.066 | 0.287 | -0.016 | 0.054 |
| ACCA | → | Anger | → | Inattention | -0.014 | 0.02 | -0.668 | 0.504 | -0.053 | 0.026 |
| ACCA | → | TR Anger | → | Inattention | -33.85 | 0.016 | -0.018 | 0.986 | -0.033 | 0.032 |
| ACCA | → | Post Control | → | Inattention | -0.021 | 0.019 | -1.127 | 0.26 | -0.058 | 0.016 |
| ACCA | → | IPS-Punishment | → | Inattention | 0.01 | 0.017 | 0.575 | 0.565 | -0.023 | 0.042 |
| *Note.*  Delta method standard errors, normal theory confidence intervals, ML estimator. | | | | | | | | | | |

**Table S10**

*Residual Covariances*

|  | | | | | | | **95% Confidence Interval** | |
| --- | --- | --- | --- | --- | --- | --- | --- | --- |
|  |  |  | **Estimate** | **Std. Error** | **z-value** | **p** | **Lower** | **Upper** |
| Anger | ↔ | TR Anger | -0.09 | 0.075 | -1.201 | 0.23 | -0.237 | 0.057 |
| Anger | ↔ | Post Control | 0.033 | 0.074 | 0.445 | 0.656 | -0.113 | 0.179 |
| TR Anger | ↔ | Post Control | -0.044 | 0.075 | -0.588 | 0.557 | -0.192 | 0.103 |
| Anger | ↔ | IPS-Punishment | -0.013 | 0.075 | -0.175 | 0.861 | -0.159 | 0.133 |
| TR Anger | ↔ | IPS-Punishment | 0.109 | 0.076 | 1.445 | 0.149 | -0.039 | 0.258 |
| Post Control | ↔ | IPS-Punishment | 0.032 | 0.075 | 0.419 | 0.675 | -0.116 | 0.179 |
| Anxiety | ↔ | Depression | 0.476 | 0.084 | 5.679 | < .001 | 0.312 | 0.64 |
| Anxiety | ↔ | Thought P. | 0.494 | 0.082 | 5.998 | < .001 | 0.333 | 0.655 |
| Depression | ↔ | Thought P. | 0.444 | 0.081 | 5.487 | < .001 | 0.285 | 0.602 |
| Anxiety | ↔ | Rule-Breaking | 0.339 | 0.079 | 4.298 | < .001 | 0.185 | 0.494 |
| Depression | ↔ | Rule-Breaking | 0.145 | 0.075 | 1.918 | 0.055 | -0.003 | 0.293 |
| Thought P. | ↔ | Rule-Breaking | 0.282 | 0.076 | 3.733 | < .001 | 0.134 | 0.43 |
| Anxiety | ↔ | Aggressivity | 0.487 | 0.084 | 5.778 | < .001 | 0.322 | 0.653 |
| Depression | ↔ | Aggressivity | 0.482 | 0.084 | 5.707 | < .001 | 0.316 | 0.648 |
| Thought P. | ↔ | Aggressivity | 0.425 | 0.08 | 5.287 | < .001 | 0.267 | 0.582 |
| Rule-Breaking | ↔ | Aggressivity | 0.463 | 0.083 | 5.566 | < .001 | 0.3 | 0.626 |
| Anxiety | ↔ | Somatic P. | 0.415 | 0.081 | 5.096 | < .001 | 0.255 | 0.575 |
| Depression | ↔ | Somatic P. | 0.394 | 0.081 | 4.854 | < .001 | 0.235 | 0.553 |
| Thought P. | ↔ | Somatic P. | 0.4 | 0.079 | 5.051 | < .001 | 0.245 | 0.555 |
| Rule-Breaking | ↔ | Somatic P. | 0.201 | 0.076 | 2.640 | 0.008 | 0.052 | 0.349 |
| Aggressivity | ↔ | Somatic P. | 0.435 | 0.083 | 5.274 | < .001 | 0.274 | 0.597 |
| Anxiety | ↔ | Social P | 0.566 | 0.087 | 6.507 | < .001 | 0.396 | 0.737 |
| Depression | ↔ | Social P | 0.497 | 0.085 | 5.870 | < .001 | 0.331 | 0.663 |
| Thought P. | ↔ | Social P | 0.52 | 0.083 | 6.233 | < .001 | 0.357 | 0.684 |
| Rule-Breaking | ↔ | Social P | 0.289 | 0.078 | 3.715 | < .001 | 0.137 | 0.442 |
| Aggressivity | ↔ | Social P | 0.513 | 0.085 | 6.007 | < .001 | 0.345 | 0.68 |
| Somatic P. | ↔ | Social P | 0.427 | 0.082 | 5.216 | < .001 | 0.267 | 0.588 |
| Anxiety | ↔ | Inattention | 0.402 | 0.082 | 4.915 | < .001 | 0.242 | 0.562 |
| Depression | ↔ | Inattention | 0.491 | 0.085 | 5.771 | < .001 | 0.324 | 0.657 |
| Thought P. | ↔ | Inattention | 0.428 | 0.081 | 5.300 | < .001 | 0.27 | 0.586 |
| Rule-Breaking | ↔ | Inattention | 0.413 | 0.082 | 5.048 | < .001 | 0.253 | 0.574 |
| Aggressivity | ↔ | Inattention | 0.542 | 0.087 | 6.225 | < .001 | 0.371 | 0.713 |
| Somatic P. | ↔ | Inattention | 0.378 | 0.081 | 4.667 | < .001 | 0.219 | 0.537 |
| Social P | ↔ | Inattention | 0.531 | 0.086 | 6.149 | < .001 | 0.362 | 0.7 |
| *Note.*  Delta method standard errors, normal theory confidence intervals, ML estimator. | | | | | | | | |

**Table S11**

*Path Coefficients*

|  | | | | | | | **95% Confidence Interval** | |
| --- | --- | --- | --- | --- | --- | --- | --- | --- |
|  |  |  | **Estimate** | **Std. Error** | **z-value** | **p** | **Lower** | **Upper** |
| Anger | → | Anxiety | -0.007 | 0.08 | -0.092 | 0.927 | -0.164 | 0.149 |
| TR Anger | → | Anxiety | -0.077 | 0.08 | -0.968 | 0.333 | -0.233 | 0.079 |
| Post Control | → | Anxiety | -0.146 | 0.079 | -1.852 | 0.064 | -0.301 | 0.008 |
| IPS-Punishment | → | Anxiety | 0.179 | 0.079 | 2.256 | 0.024* | 0.023 | 0.334 |
| ACCA | → | Anxiety | -0.011 | 0.084 | -0.13 | 0.896 | -0.175 | 0.153 |
| Anger | → | Depression | 0.001 | 0.08 | 0.018 | 0.986 | -0.156 | 0.159 |
| TR Anger | → | Depression | -0.195 | 0.097 | -2.008 | 0.045* | -0.465 | -0.013 |
| Post Control | → | Depression | -0.16 | 0.079 | -2.014 | 0.044* | -0.315 | -0.004 |
| IPS-Punishment | → | Depression | 0.062 | 0.08 | 0.777 | 0.437 | -0.094 | 0.218 |
| ACCA | → | Depression | 0.087 | 0.084 | 1.030 | 0.303 | -0.078 | 0.252 |
| Anger | → | Thought P. | 0.234 | 0.078 | 3.010 | 0.003** | 0.082 | 0.386 |
| TR Anger | → | Thought P. | -0.085 | 0.077 | -1.097 | 0.273 | -0.237 | 0.067 |
| Post Control | → | Thought P. | -0.078 | 0.077 | -1.016 | 0.31 | -0.229 | 0.073 |
| IPS-Punishment | → | Thought P. | 0.195 | 0.077 | 2.524 | 0.012* | 0.043 | 0.346 |
| ACCA | → | Thought P. | 0.127 | 0.082 | 1.562 | 0.118 | -0.032 | 0.287 |
| Anger | → | Rule-Breaking | -0.048 | 0.08 | -0.598 | 0.55 | -0.203 | 0.108 |
| TR Anger | → | Rule-Breaking | -0.093 | 0.079 | -1.170 | 0.242 | -0.248 | 0.063 |
| Post Control | → | Rule-Breaking | -0.082 | 0.079 | -1.038 | 0.299 | -0.236 | 0.073 |
| IPS-Punishment | → | Rule-Breaking | 0.18 | 0.079 | 2.275 | 0.023* | 0.025 | 0.334 |
| ACCA | → | Rule-Breaking | 0.115 | 0.084 | 1.371 | 0.17 | -0.049 | 0.278 |
| Anger | → | Aggressivity | 0.087 | 0.08 | 1.082 | 0.279 | -0.071 | 0.244 |
| TR Anger | → | Aggressivity | -0.013 | 0.08 | -0.168 | 0.867 | -0.171 | 0.144 |
| Post Control | → | Aggressivity | -0.096 | 0.08 | -1.209 | 0.227 | -0.252 | 0.06 |
| IPS-Punishment | → | Aggressivity | 0.269 | 0.107 | 2.509 | 0.012* | 0.051 | 0.464 |
| ACCA | → | Aggressivity | 0.112 | 0.084 | 1.324 | 0.185 | -0.054 | 0.277 |
| Anger | → | Somatic P. | -0.147 | 0.08 | -1.835 | 0.066 | -0.303 | 0.01 |
| TR Anger | → | Somatic P. | -0.125 | 0.08 | -1.575 | 0.115 | -0.282 | 0.031 |
| Post Control | → | Somatic P. | -0.106 | 0.079 | -1.338 | 0.181 | -0.261 | 0.049 |
| IPS-Punishment | → | Somatic P. | 0.096 | 0.079 | 1.216 | 0.224 | -0.059 | 0.252 |
| ACCA | → | Somatic P. | 0.044 | 0.084 | 0.52 | 0.603 | -0.121 | 0.208 |
| Anger | → | Social P. | 0.026 | 0.08 | 0.327 | 0.743 | -0.131 | 0.183 |
| TR Anger | → | Social P. | -0.051 | 0.08 | -0.636 | 0.524 | -0.207 | 0.106 |
| Post Control | → | Social P. | -0.251 | 0.103 | -2.430 | 0.015* | -0.529 | 0.006 |
| IPS-Punishment | → | Social P. | 0.092 | 0.079 | 1.166 | 0.244 | -0.063 | 0.248 |
| ACCA | → | Social P. | 0.162 | 0.084 | 1.932 | 0.053 | -0.002 | 0.327 |
| Anger | → | Inattention | 0.055 | 0.081 | 0.683 | 0.494 | -0.103 | 0.213 |
| TR Anger | → | Inattention | -0.001 | 0.08 | -0.018 | 0.986 | -0.159 | 0.156 |
| Post Control | → | Inattention | -0.098 | 0.08 | -1.233 | 0.218 | -0.255 | 0.058 |
| IPS-Punishment | → | Inattention | 0.047 | 0.08 | 0.589 | 0.556 | -0.11 | 0.204 |
| ACCA | → | Inattention | 0.159 | 0.085 | 1.872 | 0.061 | -0.007 | 0.325 |
| ACCA | → | Anger | -0.245 | 0.077 | -3.200 | 0.001** | -0.395 | -0.095 |
| ACCA | → | TR Anger | 0.205 | 0.077 | 2.649 | 0.008** | 0.053 | 0.357 |
| ACCA | → | Post Control | 0.214 | 0.077 | 2.772 | 0.006** | 0.063 | 0.365 |
| ACCA | → | IPS-Punishment | 0.204 | 0.077 | 2.634 | 0.008** | 0.052 | 0.356 |
| *Note.*  Delta method standard errors, normal theory confidence intervals, ML estimator. | | | | | | | | |

**Table S12**

*Path Coefficients*

|  | | | | | | | **95% Confidence Interval** | |
| --- | --- | --- | --- | --- | --- | --- | --- | --- |
|  |  |  | **Estimate** | **Std. Error** | **z-value** | **p** | **Lower** | **Upper** |
| Anxiety | → | Anger | -0.09 | 0.097 | -0.934 | 0.35 | -0.28 | 0.099 |
| Depression | → | Anger | -0.086 | 0.096 | -0.898 | 0.369 | -0.274 | 0.102 |
| Somatic P. | → | Anger | -0.252 | 0.085 | -2.958 | 0.003 | -0.419 | -0.085 |
| Thought P. | → | Anger | 0.371 | 0.092 | 4.049 | < .001 | 0.191 | 0.551 |
| Rule-Breaking | → | Anger | -0.142 | 0.088 | -1.619 | 0.105 | -0.314 | 0.03 |
| Agresivity | → | Anger | 0.159 | 0.102 | 1.558 | 0.119 | -0.041 | 0.36 |
| Inattention | → | Anger | 0.03 | 0.097 | 0.31 | 0.757 | -0.159 | 0.219 |
| ACCA | → | Anger | -0.26 | 0.072 | -3.596 | < .001 | -0.402 | -0.118 |
| Anxiety | → | TR Anger | 0.047 | 0.103 | 0.459 | 0.646 | -0.155 | 0.25 |
| Depression | → | TR Anger | -0.177 | 0.103 | -1.726 | 0.084 | -0.378 | 0.024 |
| Somatic P. | → | TR Anger | -0.086 | 0.091 | -0.94 | 0.347 | -0.264 | 0.093 |
| Thought P. | → | TR Anger | -0.059 | 0.098 | -0.599 | 0.549 | -0.251 | 0.133 |
| Rule-Breaking | → | TR Anger | -0.123 | 0.094 | -1.309 | 0.191 | -0.307 | 0.061 |
| Aggressivity | → | TR Anger | 0.123 | 0.109 | 1.127 | 0.26 | -0.091 | 0.338 |
| Inattention | → | TR Anger | 0.118 | 0.103 | 1.147 | 0.251 | -0.084 | 0.321 |
| ACCA | → | TR Anger | 0.209 | 0.077 | 2.710 | 0.007 | 0.058 | 0.361 |
| Anxiety | → | Post Control | -0.084 | 0.104 | -0.811 | 0.417 | -0.287 | 0.119 |
| Depression | → | Post Control | -0.121 | 0.103 | -1.174 | 0.241 | -0.322 | 0.081 |
| Somatic P. | → | Post Control | -0.033 | 0.091 | -0.363 | 0.717 | -0.212 | 0.146 |
| Thought P. | → | Post Control | 0.062 | 0.098 | 0.63 | 0.529 | -0.131 | 0.254 |
| Rule-Breaking | → | Post Control | -0.04 | 0.094 | -0.424 | 0.671 | -0.224 | 0.145 |
| Aggressivity | → | Post Control | 0.032 | 0.11 | 0.288 | 0.773 | -0.183 | 0.247 |
| Inattention | → | Post Control | -0.01 | 0.104 | -0.092 | 0.926 | -0.213 | 0.193 |
| ACCA | → | Post Control | 0.217 | 0.078 | 2.802 | 0.005 | 0.065 | 0.369 |
| Anxiety | → | IPS-Punishment | 0.093 | 0.102 | 0.904 | 0.366 | -0.108 | 0.293 |
| Depression | → | IPS-Punishment | -0.062 | 0.102 | -0.615 | 0.539 | -0.262 | 0.137 |
| Somatic P. | → | IPS-Punishment | 5.490×10^-4^ | 0.09 | 0.006 | 0.995 | -0.176 | 0.177 |
| Thought P. | → | IPS-Punishment | 0.147 | 0.097 | 1.511 | 0.131 | -0.044 | 0.337 |
| Rule-Breaking | → | IPS-Punishment | 0.12 | 0.093 | 1.287 | 0.198 | -0.063 | 0.302 |
| Aggressivity | → | IPS-Punishment | 0.032 | 0.108 | 0.294 | 0.769 | -0.181 | 0.244 |
| Inattention | → | IPS-Punishment | -0.104 | 0.102 | -1.019 | 0.308 | -0.305 | 0.096 |
| ACCA | → | IPS-Punishment | 0.193 | 0.077 | 2.517 | 0.012 | 0.043 | 0.343 |
| Anxiety | → | APS-Punishment | 0.045 | 0.103 | 0.44 | 0.66 | -0.156 | 0.247 |
| Depression | → | APS-Punishment | 0.043 | 0.102 | 0.424 | 0.671 | -0.157 | 0.243 |
| Somatic P. | → | APS-Punishment | -0.131 | 0.091 | -1.448 | 0.148 | -0.309 | 0.046 |
| Thought P. | → | APS-Punishment | -0.172 | 0.097 | -1.769 | 0.077 | -0.363 | 0.019 |
| Rule-Breaking | → | APS-Punishment | -0.197 | 0.093 | -2.113 | 0.035 | -0.38 | -0.014 |
| Aggressivity | → | APS-Punishment | 0.278 | 0.109 | 2.552 | 0.011 | 0.064 | 0.491 |
| Inattention | → | APS-Punishment | 0.08 | 0.103 | 0.783 | 0.434 | -0.121 | 0.282 |
| ACCA | → | APS-Punishment | -0.092 | 0.077 | -1.192 | 0.233 | -0.242 | 0.059 |
| ACCA | → | Anxiety | -0.02 | 0.079 | -0.251 | 0.802 | -0.175 | 0.135 |
| ACCA | → | Depression | 0.037 | 0.079 | 0.47 | 0.639 | -0.118 | 0.192 |
| ACCA | → | Somatic P. | 0.051 | 0.079 | 0.644 | 0.52 | -0.104 | 0.206 |
| ACCA | → | Thought P. | 0.076 | 0.079 | 0.958 | 0.338 | -0.079 | 0.23 |
| ACCA | → | Rule-Breaking | 0.126 | 0.078 | 1.611 | 0.107 | -0.027 | 0.28 |
| ACCA | → | Aggressivity | 0.093 | 0.079 | 1.187 | 0.235 | -0.061 | 0.248 |
| ACCA | → | Inattention | 0.133 | 0.078 | 1.702 | 0.089 | -0.02 | 0.287 |
| *Note.*  Delta method standard errors, normal theory confidence intervals, ML estimator. | | | | | | | | |
